# Fast and Efficient *Drosophila melanogaster* Gene Knock-Ins Using MiMIC Transposons

**DOI:** 10.1534/g3.114.014803

**Published:** 2014-10-08

**Authors:** Sven Vilain, Roeland Vanhauwaert, Ine Maes, Nils Schoovaerts, Lujia Zhou, Sandra Soukup, Raquel da Cunha, Elsa Lauwers, Mark Fiers, Patrik Verstreken

**Affiliations:** * Department of Human Genetics and Leuven Research Institute for Neuroscience and Disease (LIND), KU Leuven, 3000 Leuven, Belgium; † VIB Center for the Biology of Disease, 3000 Leuven, Belgium

**Keywords:** *Drosophila*, genome editing, homologous recombination, MiMIC

## Abstract

Modern molecular genetics studies necessitate the manipulation of genes in their endogenous locus, but most of the current methodologies require an inefficient donor-dependent homologous recombination step to locally modify the genome. Here we describe a methodology to efficiently generate *Drosophila* knock-in alleles by capitalizing on the availability of numerous genomic MiMIC transposon insertions carrying recombinogenic *attP* sites. Our methodology entails the efficient PhiC31-mediated integration of a recombination cassette flanked by unique *I-SceI* and/or *I-CreI* restriction enzyme sites into an *attP*-site. These restriction enzyme sites allow for double-strand break−mediated removal of unwanted flanking transposon sequences, while leaving the desired genomic modifications or recombination cassettes. As a proof-of-principle, we mutated *LRRK*, *tau*, and *sky* by using different MiMIC elements. We replaced 6 kb of genomic DNA encompassing the *tau* locus and 35 kb encompassing the *sky* locus with a recombination cassette that permits easy integration of DNA at these loci and we also generated a functional *LRRK^HA^* knock in allele. Given that ~92% of the *Drosophila* genes are located within the vicinity (<35 kb) of a MiMIC element, our methodology enables the efficient manipulation of nearly every locus in the fruit fly genome without the need for inefficient donor-dependent homologous recombination events.

The continued development of novel genetic tools to manipulate gene function in *Drosophila* has boosted the use of this organism to study *in vivo* biological mechanisms and to model disease (Bier 2005; Yamamoto *et al.* 2014). Ideally genes are modified in their endogenous locus by the use of homologous recombination (Rong and Golic 2000; Chan *et al.* 2012), but this process is rather tedious and inefficient. Hence, methodologies that facilitate *Drosophila* genome editing and allow for efficient repetitive targeting of a locus without the need for homologous recombination are eagerly welcomed (Gao *et al.* 2008; Choi *et al.* 2009; Huang *et al.* 2009; Weng *et al.* 2009).

Improvements in technology to target loci by homologous recombination have recently been made. These are based on generating a double-strand break (DSB) in the target DNA near or in the locus of interest and then allow manipulations via repair mechanisms, including donor dependent repair by homologous recombination. These DSBs can be introduced by nucleases, including zinc finger nucleases (ZFN) (Bibikova *et al.* 2003), TAL effector nucleases (TALEN) (Liu *et al.* 2012), or CRISPR-associated protein9 (Cas9)(Jinek *et al.* 2012; Cong *et al.* 2013; Gratz *et al.* 2013) that are designed to recognize their target DNA in a sequence-dependent manner. The ability to introduce a DSB increases the efficiency of homologous recombination by donor dependent repair by 10- to 100-fold (Rong *et al.* 2002; Bibikova *et al.* 2003). However, a given endonuclease or Cas9/gRNA pair may find promiscuous cut sites in the genome (Hsu *et al.* 2013; Mali *et al.* 2013; Pattanayak *et al.* 2013) and in addition, every locus in the genome requires the design and the testing of new nucleases (TALEN, ZFN) or guide RNA (Cas9) molecules to target the locus of interest. Finally, the identification of a modified genomic locus in the *Drosophila* genome ideally requires the integration or the excision of selection markers and such strategies often result in leaving behind recombination recognition sites (*FRT*, *LoxP*, *attR*, …) (Gao *et al.* 2008; Choi *et al.* 2009; Huang *et al.* 2009; Weng *et al.* 2009; Wesolowska and Rong 2013). These sites would then still need to be removed from the genome in an independent step. Hence, a strategy that does not require the need to test tools locus-by-locus would allow one to efficiently modify the locus of interest without the need for homologous recombination and without leaving exogenous sequences could serve as a parallel alternative to the current genome editing methodologies.

Compared with classical homologous recombination using a donor construct, homologous recombination by single strand annealing is 100-fold more efficient (Rong and Golic 2000; Rong *et al.* 2002). Single-strand annealing entails homologous recombination between two regions of homology located on the same chromosome. Hence, if a homology cassette that harbors a homology arm is provided nearby the locus of interest, a DSB flanking this cassette induces homologous recombination by single-strand annealing between the regions of homology with high efficiency (up to 85%) (Rong and Golic 2000). Hence, an alternative methodology for genome editing would (1) facilitate the integration of such a homology cassette nearby the locus of interest, using a methodology different from donor-dependent homologous recombination; and (2) use single-strand annealing to resolve this cassette, leaving mutations or other functional sequences behind.

Here we describe a methodology that allows for efficient genome editing in nearly every *Drosophila* locus without the need for donor dependent homologous recombination. Our methodology capitalizes on the ongoing efforts of the *Drosophila* Gene Disruption Project that has generated numerous MiMIC transposon insertions nearby or in many genes in the *Drosophila* genome (Venken *et al.* 2011). MiMIC transposons that carry *attP* sites allow for effective PhiC31-mediated integration of a recombination cassette that we flanked by unique restriction enzyme sites. These sites allow one to efficiently generate DSBs followed by homologous recombination by single-strand annealing, thereby locally editing the genome. We inserted an HA tag in the *LRRK* gene by using a MiMIC in a neighboring gene, and we replaced 6 kb and 35 kb encompassing the *tau* and *sky* loci, respectively, with a cassette that allows for recombination-mediated DNA exchange in these loci. The high efficiency of our approach allowed us to screen for correct modification of the loci by simple genomic polymerase chain reaction (PCR) of a limited number of individual fly lines. Given that MiMIC insertions are widely present in the fly genome (Venken *et al.* 2011), our method enables to manipulate almost every *Drosophila* gene without the need for donor-dependent homologous recombination.

## Materials and Methods

### Molecular genetics and *Drosophila* maintenance

The recombination cassette to be integrated *Mi{MIC}GluRIIE^MI01886^* was created by chimeric PCR of *LRRK* from the BAC clone CH322-120O10 with the following primers: 5′-CAG GTA CCA GTT ACG CTA GGG ATA ACA GGG TAA TAT AGG CCC AAG ATG AAC ATG TTG TGC-3′, introducing an *I-SceI* restriction site and a *Kpn*I restriction site 1095 bp upstream of the *LRRK* stop codon; 5′-CGC CAA GCA CTG GAC CTA CCC ATA CGA CGT ACC AGA TTA CGC TTA CCC ATA CGA CGT ACC AGA TTA CGC TTA CCC ATA CGA CGT ACC AGA TTA CGC T-3′ and 5′-TAC CCA TAC GAC GTA CCA GAT TAC GCT TAC CCA TAC GAC GTA CCA GAT TAC GCT TAC CCA TAC GAC GTA CCA GAT TAC GCT TAA ATT CGA TCT CAT TCA AAA TAT TTG-3′, introducing a 3xHA tag in front of the stop codon of *LRRK*; and 5′CAA TCG GCA TCC GAT AAG TGC AAA ACG TCG TGA GAC AGT TTG GTG GTA CC-3′, introducing an *I-CreI* restriction site and a *Kpn*I restriction site 639 bp downstream of the *LRRK* stop codon. This PCR product was subsequently cloned in the *pABC* plasmid with the use of restriction-ligation with *Kpn*I (Choi *et al.* 2009); *pABC* harbors two *attB* sites that allow for PhiC31-mediated integration in the *attP* sites of a MiMIC element. This plasmid was injected (*GenetiVision*, Houston, TX) in *Drosophila* embryos carrying both the *Mi{MIC}GluRIIE^MI01886^* and an embryonic source of PhiC31 (Bischof *et al.* 2007).

Positive integration events were selected by scoring for the absence of *y^+^* followed by PCR to assess the orientation of the insert using the following primers: 5′-GCG ATT GAT GAG CAT GTG AAC-3′ (forward primer the end of the *LRRK* gene) and 5′-GTT ACG CTA GGG ATA ACA GG-3′ (reverse primer in the *I-SceI* restriction site). The “targeting plasmid,” pSV001, was generated by ligating a synthetic DNA fragment carrying multiple restriction sites, an *I-SceI* recognition site, and an *F3* recombination site (Integrated DNA Technologies) in *pFL44S{w+}-attB* linearized by *Kpn*I and *Xba*I and treated with “Alkaline Phosphatase, Calf intestinal” (New England Biolabs). The sequence of the synthetic *KpnI-ISceI-AgeI-SmaI-AvrII-F3-XbaI* fragment was 5′-ATG CGG TAC CGG ATA GGG ATA ACA GGG TAA TAT AGA CCG GTC CCG GGC CTA GGG AAG TTC CTA TAC TAT TTG AAG AAT AGG AAC TTC GGA ATA GGA ACT TCT CTA GAA TGC-3′. Homology arms for *tau* were PCR amplified with following primers, *tau* (fw) 5′-CGC GAC CGG TCT AAG TGC AAC AAC GCC GAG ATT TGG-3′ (with an *Age*I site), and *tau* (rev) 5′-GCG CCC TAG GGC CGA AAT GCA TGT CGA GCT GTA TC-3′ (with an *AvrII* site) respectively and cloned into pSV001. Homology arms for *sky* were PCR amplified with the following primers: *sky* (fw) 5′-GAC TGG ATC CTA GGG ATA ACA GGG TAA TAC CGG TTC TAG ACT CGA GCG GCA GTC TGG TCT TGT TTC-3′ (with an *I-SceI* site) and *sky* (rev) 5′-GTC ACT GCA GGA AGT TCC TAT ACT ATT TGA AGA ATA GGA ACT TCG GAA TAG GAA CTT CAC TAG TGG CGC GCC AAG CTT CTA TTT CAT TCT TCT AGG GGC-3′ (with an *F3* site) and directly cloned into *pFL44S{w+}-attB*. These plasmids were injected into the following MiMICs harboring transgenic flies (BestGene Inc): *Mi{MIC}sky^MI04695^* and *Mi{MIC}tau^MI03440^*.

Positive integration events were selected by scoring for w^+^ progeny (BestGene inc) followed by PCR to assess which *attR* were generated with following primers, *I-SceI* primer: 5′-CGG-TAT TAC CCT GTT ATC CC-3′and *sky* and *tau* primers, 5′-CTG CGG CTG CAA TTT ATT TC-3′ (*sky*), 5′-GCA AGT AGG TCG CAT CGG CC-3′ (*tau*), respectively. DSBs were generated by 1 hr 37° heat shock in second instar larvae. Primers used for PCR screening after single-strand annealing are as follows: for loss of the *I-SceI* site: 5′-GTT ACG CTA GGG ATA ACA GG-3′ (reverse primer *I-SceI* restriction site) and 5′-CAC ATT CAT TGC CTG CTG TGG-3′ (forward primer *LRRK* 3′), and for loss of the *I-CreI* site: 5′-CGT CGT GAG ACA GTT TGG-3′ (reverse primer *I-CreI* restriction site) and 5′-GCG ATT GAT GAG CAT GTG AAC-3′ (forward primer end of *LRRK* gene), and finally for the integration of the HA-tag: 5′-CCA TTA GTG TTT TCC GAC C-3′ (forward primer *LRRK^HA^*) and 5′-ACT CCT CAG CGA ATA TAC C-3′ (reverse primer *LRRK^HA^*). To screen for flies that carry *sky* and *tau* deletions, PCR was performed from the novel RMCE over the duplication to the endogenous DNA with following primers: *sky* (fw) 5′-CAG AAA ACG GCG TGC GTA AG-3′ *sky* (rev) 5′-GAA TAG GAA CTT CGG AAT AGG-3′ *tau* (fw) 5′-AGG TGG CTC TGT TGG AGT TC-3′ *tau* (rev) 5′-GTT CCT ATT CCG AAG TTC CTA TTC-3′ and sequence verified. All crosses and stocks were maintained on standard cornmeal and molasses media at 25° and fly genetics and crossing schemes are shown in Supporting Information, Figure S1 and Figure S4.

### Western blot

Adult flies were decapitated with a razor blade. Heads were homogenized on ice using a motorized pellet pestle in lysis buffer containing 50 mM Tris-HCl pH 6.8, 130 mM NaCl, 1% Triton, 1 mM MgCl, and protease inhibitor complete (Roche). After a 30-min extraction on ice, the homogenate was cleared by centrifugation for 20 min at 3000 × g. Supernatant was resuspended in LDS sample buffer (Invitrogen) and denatured for 10 min at 70°. A volume of supernatant that corresponds to 15 heads was separated by sodium dodecyl sulfate polyacrylamide gel electrophoresis in 3–8% NuPAGE Tris-Acetate gels (Invitrogen) and blotted onto polyvinylidene fluoride transfer membrane (Millipore). Protein bands were visualized with a Ponceau S stain (0.1% Ponceau S and 0.5% acetic acid). Blots were blocked in TBST+5% milk and the membrane was incubated overnight with mouse anti-HA (Clone 16B12, Covance) diluted 1:500 in TBST+1% BSA or with antisynapsin [3C11 (anti-SYNORF1), Developmental Studies Hybridoma Bank, Iowa, City, IA] diluted 1:500 in TBST+1% BSA. Peroxidase-conjugated secondary antibodies and ECL plus system (Pierce) were used for detection.

### Bioinformatics

The bioinformatics analysis is based on MiMIC insertion site list (release version 27-02-2013) (Venken *et al.* 2011) and the Flybase (http://flybase.org) *Drosophila melanogaster* genome annotation version 5.51 (FlyBase Genome 2013). The analysis determined for all region sizes between 0 and 100 (with steps of 10) how many genes are either fully within the specified distance (full gene) of a MiMIC insert site, or have an overlap of at least one nucleotide (1nt). The results are combined into a total number of unique genes encompassed by or overlapping with the MiMIC region and plotted in Figure 4. The calculations were performed in an iPython (http://ipython.org/notebook.html) by use of the Pandas data analysis library (http://pandas.pydata.org/). The notebook containing the full analysis is available as a source ipynb file or a PDF (File S2).

## Results

### Direct targeting of *LRRK* using a MiMIC insertion in *GluRIIE*

**Figure 1:**
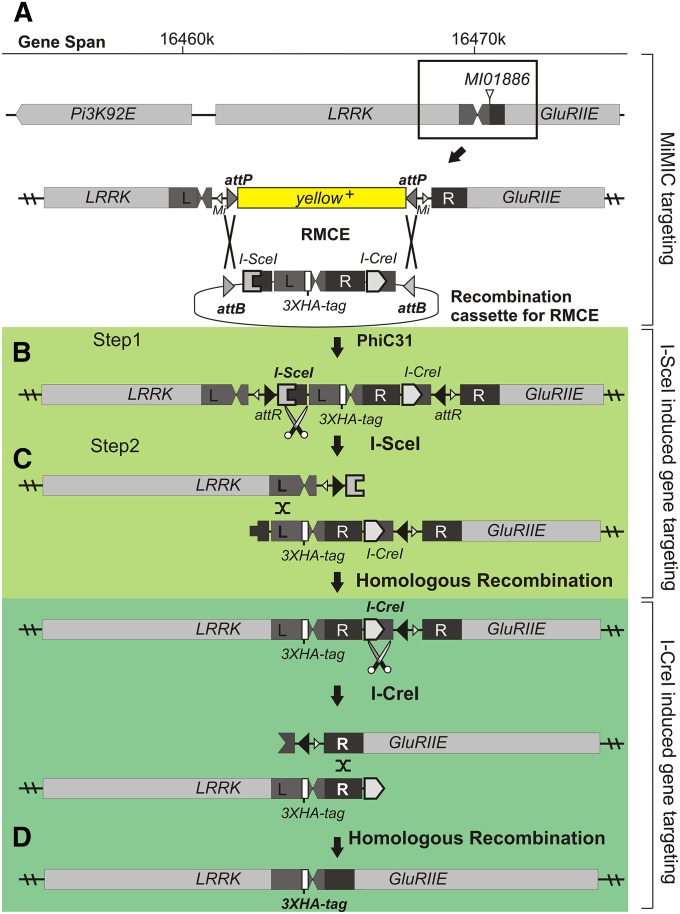
Genome editing using MiMICs through two consecutive double-strand breaks. Schematic representation of (A) gene span around the MiMIC [*Mi{MIC}GluRIIE^MI01886^* harboring a yellow^+^ marker flanked by attP sites and Minos element arms (*Mi*)] downstream of *LRRK*, which is being targeted with a targeting construct consisting of a duplication of part of *LRRK*, an HA-tag and a duplication of part of *GluRIIE* and flanked by an *I-SceI* and *I-CreI* endonuclease sites. (B) Phase 1: PhiC31-mediated integration using *attP* sites in the MiMIC and the *attB* sites of the targeting plasmid replacing the yellow^+^ marker by recombinase-mediated cassette exchange (RMCE) sequence. (C) Phase 2: two consecutive double-strand breaks, by I-SceI (light green) and I-CreI (dark green) followed by repair through single-strand annealing to remove the unwanted flanking sequences whereas (D) leaving a triple HA-tag in the endogenous *LRRK* locus. Bold text marks which sites/enzymes/mechanisms are being used. Green colors indicating I-SceI/I-CreI−induced gene targeting, and this color scheme matches that used in the crossing scheme in Figure S1.

Our methodology to manipulate the *Drosophila* genome entails a multistep process in which we first target an *attP* site in a MiMIC transposon inserted close to or in our gene of interest with a “recombination cassette” and in a second phase we resolve the cassette by single-strand annealing, thereby bridging and modifying the nearby genome while removing unwanted transposon sequences. To provide a proof of principle for our targeting methodology, we used it to knock in an HA tag in the *LRRK* gene (Figure 1). First we selected a MiMIC insertion (*Mi{MIC}GluRIIE^MI01886^*) in the *GluRIIE* gene located 3′ of the *LRRK* gene (Figure 1A). Second, we generated a recombination cassette that consists of two 500-bp stretches of sequence; one that encompasses the 3′ end of the *LRRK* gene (dark gray in Figure 1A, marked by ‘L’) and one identical to the sequence immediately 3′ of the MiMIC insertion site (black in Figure 1A, marked by ‘R’). In this cassette we inserted a sequence coding for an HA tag in front of the *LRRK* stop codon and flanked the construct with an *I-SceI* and an *I-CreI* endonuclease site as well as with *attB* sites on either side (Figure 1A, methods). This recombination cassette was injected into embryos that harbor *Mi{MIC}GluRIIE^MI01886^* and that express PhiC31 recombinase. PhiC31-mediated recombination between the *attB* sites in the recombination cassette and *attP* sites in the MiMIC replaced the *yellow^+^*(*y^+^*) marker in the MiMIC with the *I-SceI* and *I-CreI* flanked recombination cassette. Consistent with previous reports on recombination mediated cassette exchange in different contexts (Bateman *et al.* 2006; Venken *et al.* 2011), this first step of our methodology was very efficient and about 20% of the injected animals integrated the recombination cassette. Using genomic PCR we verified the orientation of the cassette and 50% of them are inserted with the *LRRK*-homology arm in the recombination cassette oriented toward the *LRRK* gene (*i.e.*, 1/10 of the injected animals; Figure 1B). Hence, using a single set of germ line injections (100 embryos) we correctly integrated the recombination cassette in *Mi{MIC}GluRIIE^MI01886^*.

**Figure 2:**
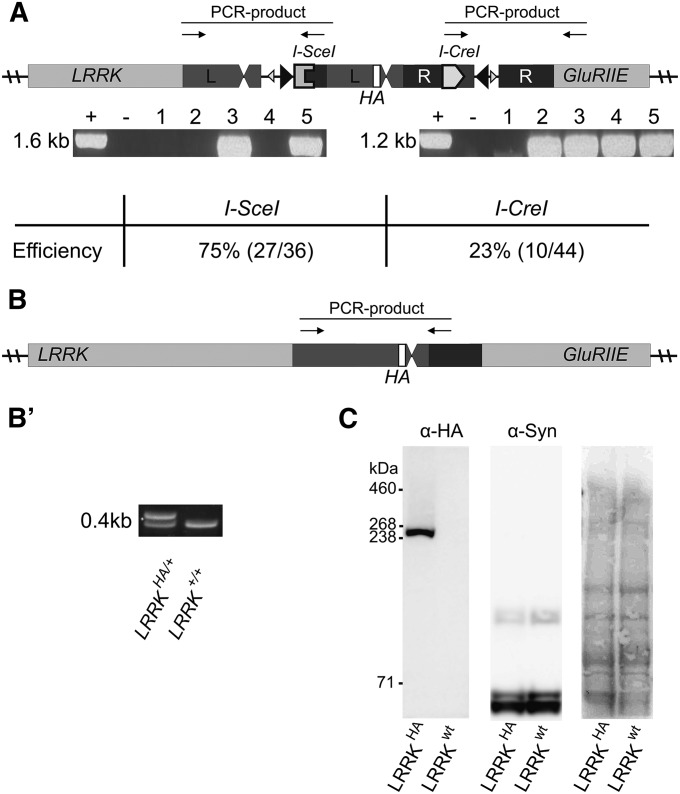
LRRK^HA^ is expressed and functional. (A) Efficiency of the two heat-shock-induced endonuclease events (*I-SceI* and *I-CreI*) used to induce double-strand breaks, assessed by PCR using primers over the *I-SceI* site and in *LRRK* as well as using primers over *I-CreI* and in *GluRIIE*. When the *I-SceI* or *I-CreI* site is absent, no PCR product can be formed. (B) Schematic representation showing where primers anneal to generate PCR product over the introduced HA-tag and (B′) PCR products, the higher product indicates the presence of the triple HA tag, the lowest band indicates the product without the tag. (C) Western blot using HA antibody showing a 250-kDa large band of LRRK^HA^ and using anti-synapsin antibody as a loading control. Right: Ponceau Red staining of the same blot.

In a second phase we resolve the transposon sequence while leaving an HA tag in the *LRRK* gene (Figure 1C). In second instar larvae we generated a DSB adjacent to the recombination cassette using I-SceI that is expressed under control of a heat inducible promotor (Figure 1C). Repair of the DSB by recombination between the regions of homology removes the 5′ transposon sequence, while leaving the HA tag in *LRRK*. To assess the efficiency of this event we screened 36 individual lines by PCR. In 27 of these lines, the transposon sequence was lost and an HA tag was inserted in the *LRRK* locus (Figure 2A). We also confirmed the presence of the HA tag by sequencing. Next, we used single-strand annealing to remove the remainder of the 3′ *MiMIC* sequence in one of the HA-tagged *LRRK* lines and expressed I-CreI under control of a heat inducible promotor (Figure 1C). PCR screening of 44 individual lines revealed that the 5′ *MiMIC* sequence was removed in 10 lines (Figure 2A), suggesting that in this experiment, I-SceI−mediated single strand annealing was somewhat more efficient than I-CreI−mediated single strand annealing (Rong and Golic 2000; Rong *et al.* 2002). PCR and sequencing of the locus revealed no aberrations except for the insertion of an HA-tag at the 3′ end of the *LRRK* open reading frame (Figure 1D and Figure 2B and B′). Hence, these single-strand annealing steps allowed us to efficiently create a knock in allele without leaving any exogenous sequence (*e.g.*, *FRT*, *LoxP*, *attR*…) in the locus (Figure 2B). The detailed crossing scheme we employed is also presented in Figure S1. All *LRRK^HA^* lines that we generated were viable, suggesting that the procedure did not induce second-site lethal aberrations.

To determine whether LRRK-HA is expressed, we used Western blotting of whole head extract of homozygous *LRRK^HA^* animals and probed the blots with anti-HA antibodies. Although we detected a clear band at 250 kDa that corresponded to LRRK-HA, this band was undetectable in control animals (Figure 2C). To further determine whether *LRRK^HA^* acts as a wild-type *LRRK* allele and recapitulates wild-type LRRK function, we also assessed synaptic vesicle endocytosis efficiency and measured the activity of the flies by using an automated monitoring system. Whereas *LRRK^P1^* mutant flies show reduced synaptic vesicle endocytosis and activity defects, *LRRK^HA^* knock in animals are indistinguishable from controls (Figure S2, A−C, File S1). Hence, at this level of analysis, synaptic function is not disrupted in *LRRK^HA^* animals, and the *LRRK^HA^* knock-in allele we generated is functional.

### Targeting *sky* and *tau* with an RMCE cassette using a MiMIC

**Figure 3:**
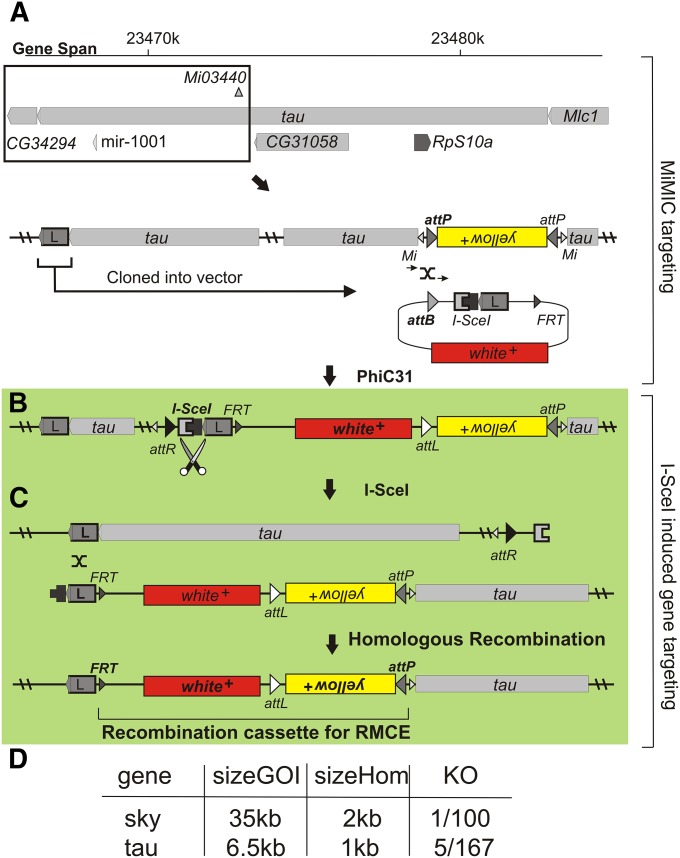
Targeting *tau* with an recombinase-mediated cassette exchange (RMCE) cassette using a MiMIC element. (A) Targeting one *attP* site in the MiMIC (*Mi{MIC}tau^MI03440^* harboring a yellow^+^ marker flanked by attP sites and Minos element arms (*Mi*)) using PhiC31-mediated integration with the pSV001 plasmid containing 5′- an attB site, an *I-SceI* restriction site, a homology arm with a stretch of DNA downstream of *tau*, an *FRT* site and a *w^+^* marker -3′. (B) I-SceI expression introduces double-strand breaks and repair (green). (C) Repair removes part of *tau* locus leaving an RMCE cassette that in a later step can be used to replace the locus with different DNA sequences. (D) Efficiencies of our approach when targeting *sky* and *tau* genes, size GOI (gene of interest) indicates the size of the deletion generated, size Hom (size homology arm) indicates the length of the used homology arm and KO (knock-out) indicates the amount of deletions per number of flies screened. Bold text marks which sites/enzymes are being used. Green color indicating I-SceI−induced gene targeting and the color scheme matches that used in Figure S4.

Next we expanded on our methodology and targeted two additional genes, *tau* and *sky*. We also adapted our method and replaced these loci with a recombination-mediated-cassette-exchange (RMCE) module. The knock-in of such a module would, in a later phase, facilitate the very efficient integration of multiple modified DNA sequences in these loci. This adapted strategy builds on the methodology we used to create *LRRK^HA^* but rather than targeting both *attP* sites in the MiMIC element to replace the *y^+^* marker, we are now targeting only one *attP* site to integrate a “targeting plasmid.” This plasmid harbors an *I*-*SceI*−flanked 1 or 2 kb homology arm identical to the sequence adjacent to the region we wish to delete, an FRT site and a *white^+^* (*w^+^*) eye marker (*tau*: Figure 3, A and B and *sky*: Figure S3A). The integration of this plasmid into the MiMIC elements using a genomic source of PhiC31 is very efficient. PCR verification indicates that for the MiMIC in *tau*, seven integrations were in the correct *attP* (the one proximal to the region duplicated in the 500 bp homology arm; Figure 3B); seven integrations occurred in both *attP* sites, and four were in the wrong *attP* site. Although for the MiMIC in *sky*, PCR verification indicates seven integrations were in the correct *attP*; 10 integrations occurred in both *attP* sites, and two were in the wrong *attP* site.

In a next step, we expressed *I-SceI* under a heat-inducible promotor in flies where the “targeting plasmid” is inserted in the correct *attP* site, thereby inducing a DSB (Figure 3C and Figure S3). The subsequent recombination event deletes the region of interest but leaves an *FRT*-*attP*−flanked RMCE cassette marked by the *w^+^* and *y^+^* visible markers. As indicated in Figure 3D and Figure S3B, our methodology to create these alleles of *tau* and *sky* was efficient, deleting 35 kb of *sky* and 6.5 kb of *tau* (Figure 3D) while replacing the deleted region with an RMCE module that will allow, in a later step, to insert modified genes at these loci. Hence, the combination of the efficient PhiC31-enabled integration of a homology arm in a MiMIC element close to a site of interest, together with restriction endonuclease-induced single strand annealing makes for a very powerful combination that allows us to manipulate and edit the genome at high efficiency over relatively long distances. A detailed crossing scheme is give in supplemental Figure S4.

### The MiMIC insertion collection permits to target most genes in the *Drosophila* genome

**Figure 4:**
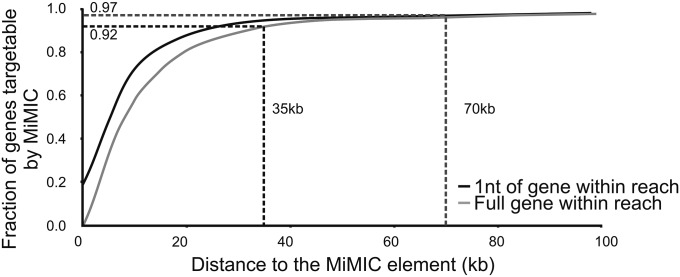
MiMICs allow the targeting of the majority of the *Drosophila* genes. Graph of distance to the MiMIC elements (kb) in function of fraction of genes targetable by a given MiMIC. Black line represents targetable genes where one nucleotide of the gene is within reach or gray line where the full gene is within reach. The dashed line at 35 kb distance to a MiMIC indicates that 92% of the *Drosophila* genes are within this distance to a MiMIC, whereas the dashed line at 70 kb indicates that 97% of the *Drosophila* genes are within this distance and within reach to perform single strand annealing (or one-ended invasion crossover) experiments (Wesolowska and Rong 2013).

To determine how widely applicable this methodology would be, we calculated the number of genes located in regions of increasing size around the MiMIC insertions currently present in the *Drosophila* genome (Venken *et al.* 2011) (6507 insertions in release version 2014-06-13). A gene is determined to be in the vicinity of a MiMIC insertion site either by the most proximal nucleotide of the gene to the MiMIC insertion site (1nt in Figure 4) or by the most distal nucleotide of the gene (full gene in Figure 4). Assuming our methodology allows to efficiently bridge 35 kb, we find that 92% of the *Drosophila* genes are within this vicinity of a MiMIC insertion site (Figure 4). Hence, the methodology we describe can be used as an alternative to existing genome editing methodologies to modulate almost all loci in the fly genome.

## Discussion

In this work, we provide proof of concept for a genome editing methodology that does not require homologous recombination by donor dependent repair and attains efficacies that are high enough to screen editing events by PCR using genomic DNA of a reasonable number of individuals. The high efficiency of genome targeting that we achieve stems from (1) the ability to insert a recombination cassette into a MiMIC transposon close to a gene of interest using PhiC31-mediated integration, and (2) resolving genomic sequence flanking the *MiMIC* insertion site using DSBs induced by I-SceI and/or I-CreI endonucleases. The power of the methodology further rests on the ever expanding collection of MiMIC transposon insertions (Venken *et al.* 2011) that harbor *attP* sites that can be used to insert a recombination cassette close to almost any *Drosophila* gene.

We replaced a region of up to 35 kb by an RMCE cassette or directly targeted a gene and introduced an HA-tag sequence using MiMICs in combination with single strand annealing or one-ended invasion crossover. Using the bridging of 35 kb as a cut off, we find that 92% of the genes are close enough to a MiMIC to be targeted using our strategy. However, in theory, single strand annealing or one-ended invasion crossover is still efficient over a 70-kb region (Wesolowska and Rong 2013) and using 70 kb as a cut off, 97% of all genes should be targetable using our strategy (Figure 4). These calculations indicate that our genome manipulation methodology is able to target most genes in the *Drosophila* genome. If the starting MiMIC insertion line does not harbor deleterious lesions, our methodology also allows us to edit the genome with very low risk of off-site genomic alterations.

Genome editing requires the insertion of exogenous DNA in the genome. Here, we used the very efficient PhiC31 integrase to insert a recombination cassette close to the locus of interest. Likewise, donor dependent homologous recombination-based methodologies also enable the insertion of exogenous DNA at the target locus, but the classical methodology is inefficient (Rong and Golic 2000; Rong *et al.* 2002). The recent development of sequence specific nucleases for the creation of DSBs at defined locations in the genome (CRISPR, TALEN, ZFN) (Bibikova *et al.* 2002; Liu *et al.* 2012; Gratz *et al.* 2013) significantly improve on the efficiency of donor dependent repair. Indeed direct editing by CRISPR/Cas9-mediated homologous recombination should permit genome modification in two generations (~1 month) (Figure S5A, dark pink). However there are two drawbacks to consider when using this direct approach: (1) the efficiency of direct targeting may not be high enough such that screening of successful events by PCR is not trivial. Integration of a visible marker would make this screening step easier, but removing this marker in a later step will add several generations of crosses (Figure S5, A and B). (2) When aiming to generate an allelic series, the direct strategy necessitates individual modifications of the genome per allele and will lead to a series of mutants that are not in an isogenic background. This is not an issue when first integrating an *attP* and creating an isogenic line that is then used to convert the attP site with the different alleles (Figure S5B) or when using the MiMIC methodology we describe. Hence, CRISPR/Cas9-mediated targeting usually starts by integrating *attP* sites with selection markers and then integrates DNA at these locations using the PhiC31 integrase (Gratz *et al.* 2013; Gratz *et al.* 2014). When including this extra “attP step,” CRISPR/Cas9 and the MiMIC-based targeting are rather similar in the time needed to create knock in alleles (compare Figure S4 and Figure S5B).

After initial targeting, both the MiMIC-based method and attP-CRISPR/Cas9 leave exogenous vector and/or marker sequence behind, and we provide a methodology to remove the unwanted vector and marker sequences that are present following the initial targeting event. Similar to the method described in Wesolowska and Rong (2013), we made use of single-strand annealing (or one-ended invasion crossover) to target genes close to an *attP* site (in our case the *attP* is provided in the MiMIC), but we postulate that this strategy can also be used when resorting to the attP-mediated CRISPR/Cas9 methodology. There are, however, also some differences. First, the method we describe here to generate an RMCE knock-in allele requires only the cloning of relatively small stretches of DNA (the homology arms); in (Wesolowska and Rong 2013) the entire locus was cloned. Of course, once the gene is targeted with an RMCE cassette and wild type or mutant genes need to be introduced, the entire locus needs to be cloned and manipulated in the methodology we describe here as well. However, these manipulations could be done in parallel with the gene targeting steps, thus saving significant amounts of time.

Second, in the method we describe to generate an RMCE knock-in, the homology arm is the only DNA stretch identical to the endogenous locus and homologous recombination after I-SceI (or I-CreI)−induced DSB can only occur between the homology arm and the endogenous locus, thereby always deleting the DNA between the MiMIC insertion site and the region identical to the homology arm. In Wesolowska and Rong (2013), homologous recombination is possible throughout the length of the construct, and it is thus possible that these recombination events fail to target the gene of interest, lowering efficiency. Hence, our method builds on but is also different from pre-existing single strand annealing methods. In addition, we have prepared a plasmid harboring *FRT(F3)* and *attB* sites that allows for RMCE with our RMCE knock-in and this plasmid is available upon request. Recent work (Chan *et al.* 2013) suggests that these single-strand annealing (or one-ended invasion crossover) steps can be shortened in time as well and the I-SceI and I-CreI steps could potentially be combined into a single step.

## Supplementary Material

Supporting Information
