# Supplementary material for: Fast and Efficient Drosophila melanogaster Gene Knock-Ins Using MiMIC Transposons
Source: G3 (Bethesda). 2014 Oct 8;4(12):2381–7. doi: 10.1534/g3.114.014803 (PMC4267933; doi:10.1534/g3.114.014803)
Supplement: Supporting Information [file supp_4_12_2381__index.html]

Fast and Efficient Drosophila melanogaster Gene Knock-Ins Using MiMIC Transposons — Supporting Information 

# Fast and Efficient *Drosophila melanogaster* Gene Knock-Ins Using MiMIC Transposons

## Supporting Information for Vilain *et al.*, 2014

**Files in this Data Supplement:**

- Supporting Information - Files S1-S2 and Figures S1-S5 (PDF, 1 MB)
- File S1 - Supporting Materials and Methods (PDF, 114 KB)
- File S2 - Bioinformatics protocol to generate Figure 4. (PDF, 255 KB)
- Figure S1 - Overview of the crossing scheme used to create double strand breaks using restriction endonucleases I-SceI and I-CreI. (PDF, 345 KB)
- Figure S2 - *LRRKHA* recapitulates wild type *LRRK* function. (PDF, 431 KB)
- Figure S3 - Strategy for *sky* targeting and molecular confirmation of targeted *sky* and *tau* loci. (PDF, 439 KB)
- Figure S4 - Crossing scheme for direct targeting of a gene of interest (here on the 2nd chromosome) using a MiMIC transposon. (PDF, 336 KB)
- Figure S5 - Crossing scheme for gene targeting using CRISPR/Cas9. (PDF, 258 KB)
