# Supplementary material for: Fast and Efficient Drosophila melanogaster Gene Knock-Ins Using MiMIC Transposons
Source: G3 (Bethesda). 2014 Oct 8;4(12):2381–7. doi: 10.1534/g3.114.014803 (PMC4267933; doi:10.1534/g3.114.014803)
Supplement: Supporting Information [file supp_g3.114.014803_014803SI.pdf]

G3·Genes | Genomes | Genetics

Fast and efficient *Drosophila melanogaster* gene knock-ins using MiMIC transposons

Sven Vilain*,§,1, Roeland Vanhauwaert*,§,1, Ine Maes*,§, Nils Schoovaerts*,§, Lujia Zhou*,§, Sandra Soukup*,§, Raquel da Cunha*,§, Elsa Lauwers*,§, Mark Fiers§ and Patrik Verstreken*,§,2

* KU Leuven, Department of Human Genetics and Leuven Research Institute for Neuroscience and Disease (LIND), 3000 Leuven, Belgium

§ VIB Center for the Biology of Disease, 3000 Leuven, Belgium

[1] These authors contributed equally to this work.

[2] Corresponding author

DOI: 10.1534/g3.114.014803

File S1

SUPPORTING MATERIALS AND METHODS

FM 1-43 Staining

FM 1-43 labeling and quantification of intensities was performed as described, using a 1 min 90 mM KCl stimulation protocol in larval boutons {Verstreken, 2008}. Upon stimulation, FM 1-43 is internalized into newly formed synaptic vesicles and its fluorescence yields a quantitative measure of synaptic vesicle formation during stimulation {Verstreken, 2008 #20}. Images were captured with a Nikon FN1 microscope and 63× 1.0 NA Water lens.

Locomotor activity analysis

3–7-day-old male flies where placed individually in glass tubes (length, 65 mm; inside diameter, 3 mm) containing 1% agar and 5% sucrose at 25 °C. Locomotor activity was monitored for 24 hours by recording infrared beam crossings by individual flies in 1 min bins using the *Drosophila* activity monitoring system (Trikinetics). Data was analyzed in Excel and statistical analysis was done with one-way ANOVA with Tukey's post hoc test, *, $p < 0.05$ for n more than 30 flies for each genotype.

File S2

Bioinformatics protocol to generate Figure 4.

Available for download at http://www.g3journal.org/lookup/suppl/doi:10.1534/g3.114.014803/-/DC1

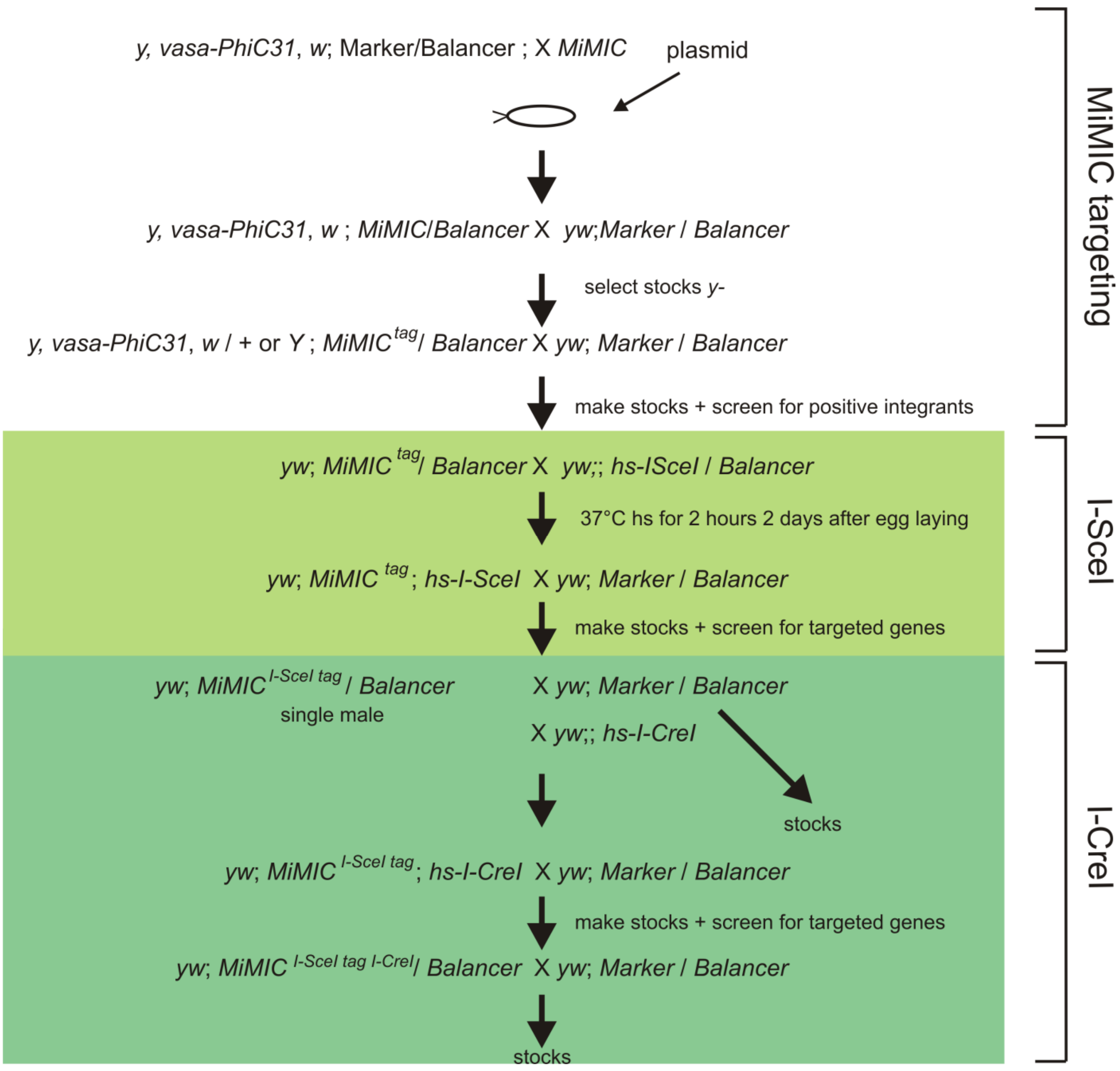

Figure S1 Overview of the crossing scheme used to create double strand breaks using restriction endonucleases I-SceI and I-CreI. During the MiMIC targeting step, the targeting plasmid is injected in embryos from flies bearing the MiMIC of interest (here for example on the 2nd chromosome) and the PhiC31 integrase. After crossing out these mosaic flies, screening for loss of yellow indicates the integration of the targeting construct in the MiMIC site. Next a stock is established and screening by PCR for integrants in the correct orientation is done. Upon I-SceI expression (light green) by crossing the *yw; MiMIC^{tag}/Balancer* stock to flies expression I-SceI under a heat shock promotor, a DSB will be generated after given a heat shock, followed by repair. After crossing out these mosaic flies the next generation will yield males that are non mosaic, since I-SceI is very efficient these males can be either used to set up stocks first or crossed simultaneous with flies that express I-CreI under heat shock promotor and flies that allow us to set up stocks. Upon I-CreI expression a DSB will be generated after a heat shock treatment, followed by repair. After crossing out these mosaic flies stocks can be set and screened for targeted genes by PCR. (dark green) (every arrow is one generation)

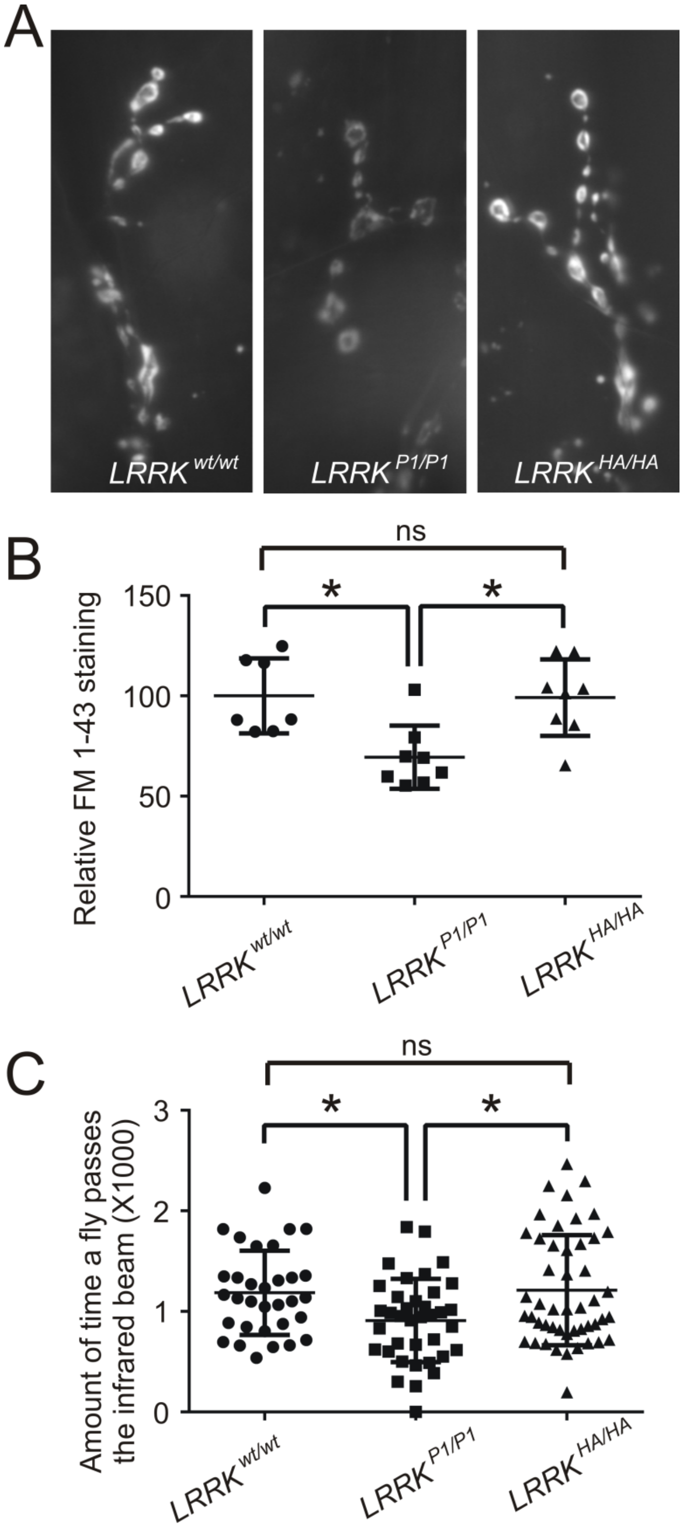

Figure S2 *LRRK*^{HA} recapitulates wild type *LRRK* function. (A) FM 1-43 staining following a 1 min 90 mM KCl stimulation protocol in larval boutons of wild type *LRRK* (*LRRK*$^{wt/wt}$), mutant *LRRK* (*LRRK*$^{P1/P1}$) and HA-tagged *LRRK* (*LRRK*$^{HA/HA}$), (B) indicating a defect in FM 1-43 dye uptake in mutant *LRRK* ^{P1} but not in HA-tagged *LRRK*^{HA} compared to wild type. Thereby showing reduced synaptic vesicle endocytosis in *LRRK* ^{P1}, not present in HA-tagged *LRRK*^{HA}. (C) Trikinetics activity assay indicating total activity counts over a 24 h period of the same genotypes as in (A) en (B). A defect in total activity in mutant *LRRK* ^{P1} but not in HA-tagged *LRRK*^{HA} compared to wild type *LRRK* is evident.

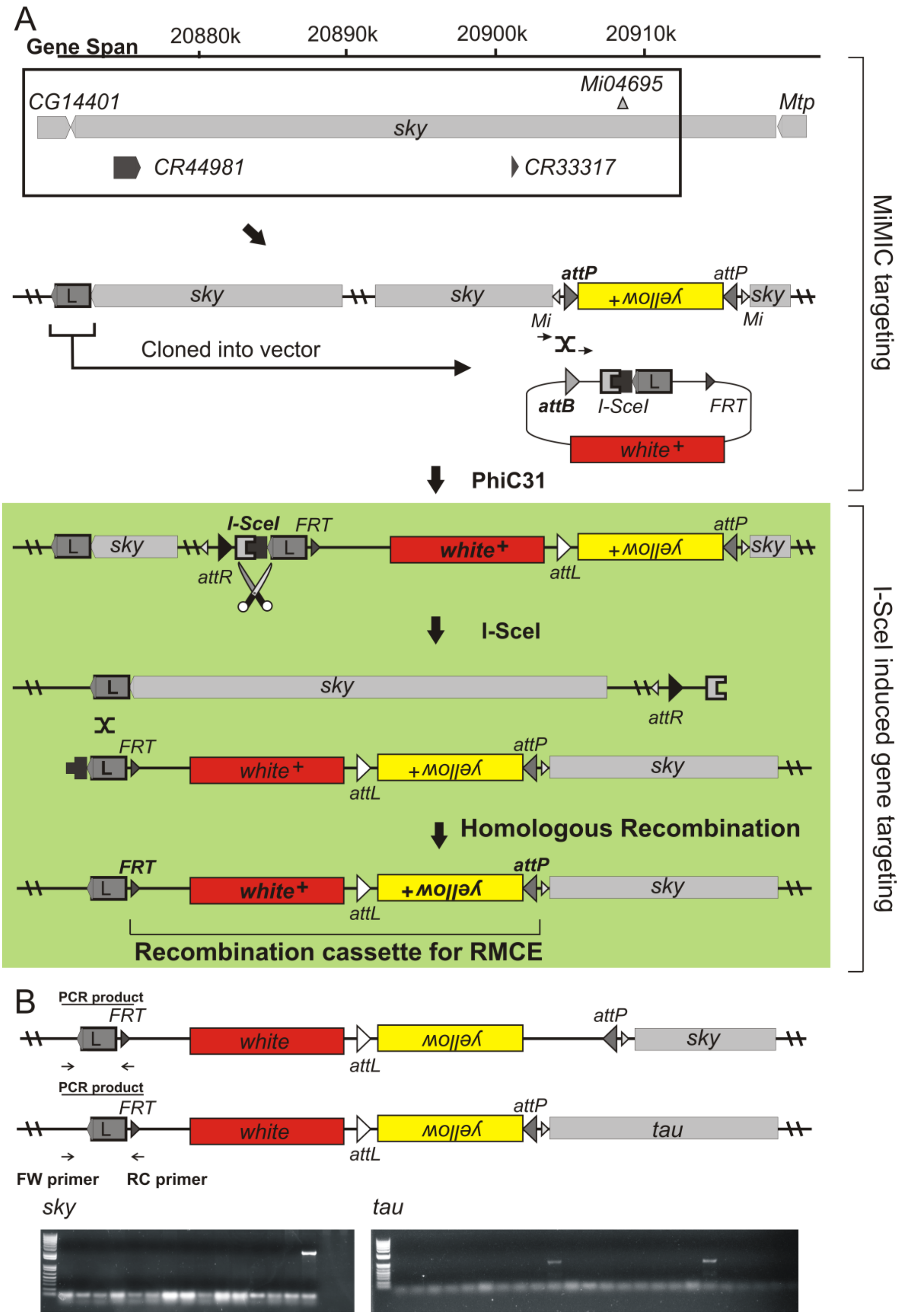

Figure S3 Strategy for *sky* targeting and molecular confirmation of targeted *sky* and *tau* loci. (A) Targeting strategy *sky* with an RMCE cassette using a MiMIC in *sky*. *I-SceI* expression followed by ssa results in a partial replacement of *sky* with an RMCE thereby deleting the majority of the sky coding sequences (green). (B) Schematic representation showing where primers (arrows) anneal to generate PCR products, one primer anneals outside the homology arm, the other anneals in the RMCE cassette. Representative agarose gels are shown, a PCR band indicates a successful targeting. Bold text marks which sites/enzymes/mechanisms are being used. Green color indicating I-SceI induced gene targeting match in crossing scheme (Figure S4).

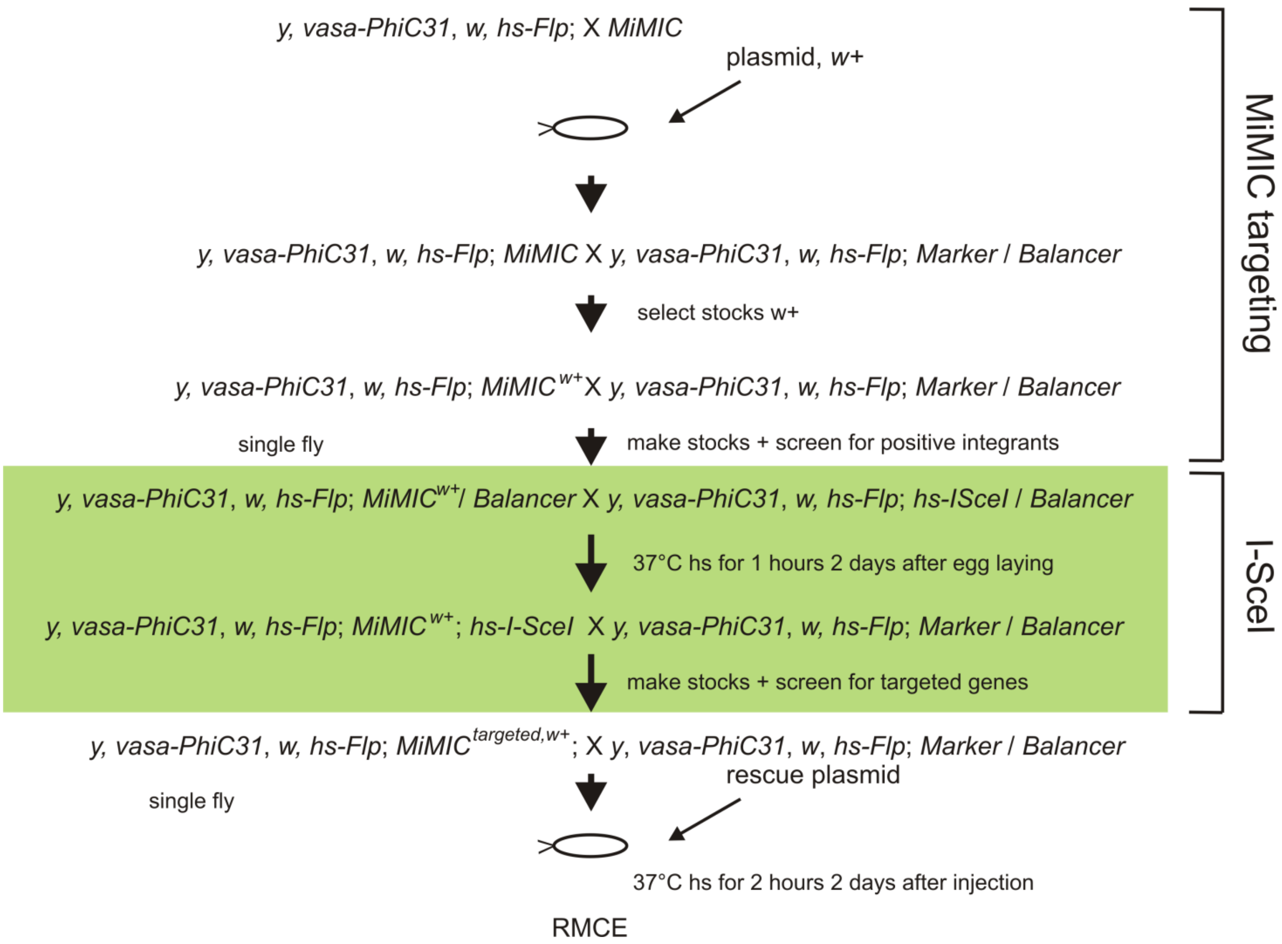

Figure S4 Crossing scheme for direct targeting of a gene of interest (here on the 2nd chromosome) using a MiMIC transposon. During the MiMIC targeting step, the targeting plasmid is injected in embryos from flies bearing the MiMIC of interest and the PhiC31 integrase. After crossing out these mosaic flies, screening for w^+ indicates the integration of the targeting construct in the MiMIC site. Next a stock is established and screening by PCR for integrants in the correct orientation is done. Crossing the *yw; MiMIC*$^{w+}$*/Balancer* stock to flies expressing I-SceI under a heat inducible promotor allows us to generate a DSB after heat shock, followed by repair. After crossing out these mosaic flies a stock is made from the offspring and screened by PCR for targeted events (green). Positive stocks replaced the gene of interest by an RMCE cassette, which in a next step can be used to introduce any construct of choice including rescue constructs via PhiC31 integration and Flp. Arrows indicate one generation.

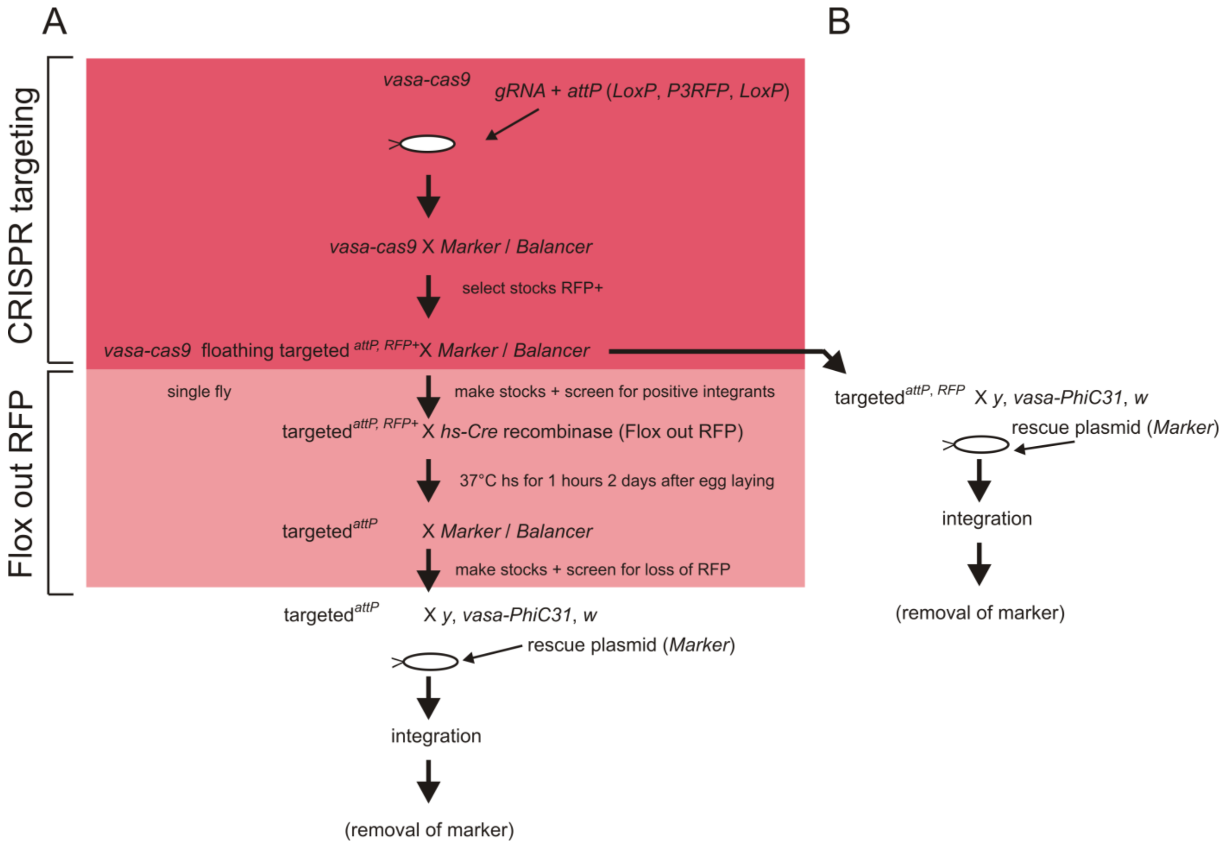

Figure S5 Crossing scheme for gene targeting using CRISPR/Cas9. (A) CRISPR targeting by injecting gRNA and the targeting construct bearing an attP site and P3 RFP marker flanked by LoxP sites, in vasa-Cas9 embryos, followed by crossing out mosaic flies and selection for RFP (dark pink). Next the RFP marker can be floxed out by crossing the targeted flies to flies expressing the Cre recombinase under a heat shock promotor, followed by crossing out mosaic flies and screening for the loss of RFP (light pink). Stocks that lost the RFP marker, bearing the attP site can in a next step be targeted with a rescue plasmid (preferentially with a marker) via PhiC31 integration. (B) Or after CRISPR targeting, single flies can be targeted with a rescue plasmid (preferentially with a marker) without first removal of the RFP marker, however these markers need to be removed afterwards. Arrows indicate one generation.
