# Supplementary material for: Fast and Efficient Drosophila melanogaster Gene Knock-Ins Using MiMIC Transposons
Source: G3 (Bethesda). 2014 Oct 8;4(12):2381–7. doi: 10.1534/g3.114.014803 (PMC4267933; doi:10.1534/g3.114.014803)
Supplement: Supporting Information [file supp_g3.114.014803_FileS1.pdf]

File S1

SUPPORTING MATERIALS AND METHODS

FM 1-43 Staining

FM 1-43 labeling and quantification of intensities was performed as described, using a 1 min 90 mM KCl stimulation protocol in larval boutons {Verstreken, 2008}. Upon stimulation, FM 1-43 is internalized into newly formed synaptic vesicles and its fluorescence yields a quantitative measure of synaptic vesicle formation during stimulation {Verstreken, 2008 #20}. Images were captured with a Nikon FN1 microscope and 63× 1.0 NA Water lens.

Locomotor activity analysis

3–7-day-old male flies where placed individually in glass tubes (length, 65 mm; inside diameter, 3 mm) containing 1% agar and 5% sucrose at 25 °C. Locomotor activity was monitored for 24 hours by recording infrared beam crossings by individual flies in 1 min bins using the *Drosophila* activity monitoring system (Trikinetics). Data was analyzed in Excel and statistical analysis was done with one-way ANOVA with Tukey's post hoc test, *, $p < 0.05$ for n more than 30 flies for each genotype.
