# Supplementary material for: Fast and Efficient Drosophila melanogaster Gene Knock-Ins Using MiMIC Transposons
Source: G3 (Bethesda). 2014 Oct 8;4(12):2381–7. doi: 10.1534/g3.114.014803 (PMC4267933; doi:10.1534/g3.114.014803)
Supplement: Supporting Information [file supp_g3.114.014803_FileS2.pdf]

**Fast and efficient *Drosophila melanogaster* gene knock-ins using MiMIC transposons**

Sven Vilain*[,§,1], Roeland Vanhauwaert*[,§,1], Ine Maes*[,§], Nils Schoovaerts*[,§], Lujia Zhou*[,§], Sandra Soukup*[,§], Raquel da Cunha*[,§], Elsa Lauwers*[,§], Mark Fiers[§] and Patrik Verstreken*[,§,2]

* KU Leuven, Department of Human Genetics and Leuven Research Institute for Neuroscience and Disease (LIND), 3000 Leuven, Belgium

[§] VIB Center for the Biology of Disease, 3000 Leuven, Belgium

[1] These authors contributed equally to this work.

[2] Corresponding author

SUPPORTING MATERIAL

Protocol S1: Bioinformatics protocol to generate Figure 1.

# 1 Analysis of all genes within 70k of a MiMIC

Mark Fiers

Version: June 2014

This document is created with iPython (http://ipython.org/) and makes use of the Python Pandas (http://pandas.pydata.org/) and Matplotlib (http://matplotlib.org/) libraries.

## 1.1 Approach

The analysis starts with a list of MiMIC elements and a list of genes. Subsequently, we check how many genes fall within a predefined range around each MiMIC element. We search for two types of overlap: one requires a gene to completely fall within the range; the other requires a gene to overlap with at least one nucleotide.

## 1.2 Load python libraries

In [23]:
```
#Pandas dataframes
import pandas as pd
#Graphs
import matplotlib.pyplot as plt
```

## 1.3 Download the MiMIC list

The following list was downloaded: http://flypush.imgen.bcm.tmc.edu/pscreen/files/MI-list-2014-06-13.xls and converted this (using openoffice) to tsv. A minor adaptation of the column names was applied for ease of processing.

This list has 6507 entries.

In [9]:
```
#milist = pd.read_csv("MiList.csv", sep="₩t")
milist = pd.read_csv("MI-list-2014-06-13.csv", sep="₩t")

# clean up column names
milist.columns = [x.lower().strip() for x in milist.columns]

# extract location into a separate chromosome and position field
splitup = milist['location'].str.split(':')
milist['chrom'] = splitup.str[0]
```

Extract position information from the list of MiMIC elements:

In [10]:
```
def get_pos(x):
    s = x.split('[')
    p = s[0].strip()
    o = s[1].replace(']', '').strip()
```

```
        try:
            return int(p), o
        except:
            return -1, o

    _pos, _ori = zip(*splitup.str[1].map(get_pos))
    milist['pos'] = _pos
    milist['orient'] = _ori

    print "Removing %d entries with unspecified positions" % len(milist[milist['pos'] == -1])
    milist = milist[milist['pos'] != -1]
    print "Kept %d records" % len(milist[milist['pos'] != -1])
```

```
Removing 139 entries with unspecified positions
Kept 6367 records
```

```
In [11]: # making a few shortcuts for later
         mchrom = milist['chrom'] #shortcut for later
         mpos = milist['pos'] #and another one

         # create two emtpy fields to contain the genes and CDSs
         # within 70k of each element
         milist['gene_in70k'] = [set() for i in range(len(milist))]
         milist['cds_in70k'] = [set() for i in range(len(milist))]
```

## 1.4 Load gene annotations

The gene annotation is available as a big GFF file from Flybase. Used version 5.50.
The file was downloaded using:

```
In [45]: !wget ftp://ftp.flybase.net/genomes/Drosophila_melanogaster/dmel_r5.57_FB2014_03/gff/dmel-all-
```

```
--2014-06-17 10:33:21--  ftp://ftp.flybase.net/genomes/Drosophila_melanogaster/dmel_r5.57_FB2014_03/gff/
           => 'dmel-all-r5.57.gff.gz'
Resolving ftp.flybase.net... 149.165.226.140
Connecting to ftp.flybase.net|149.165.226.140|:21... connected.
Logging in as anonymous ... Logged in!
==> SYST ... done.    ==> PWD ... done.
==> TYPE I ... done.  ==> CWD (1) /genomes/Drosophila_melanogaster/dmel_r5.57_FB2014_03/gff ... done.
==> SIZE dmel-all-r5.57.gff.gz ... 377738814
==> PASV ... done.    ==> RETR dmel-all-r5.57.gff.gz ... done.
Length: 377738814 (360M) (unauthoritative)

100%[======================================>] 377,738,814 3.06MB/s   in 2m 1s

2014-06-17 10:35:24 (2.98 MB/s) - 'dmel-all-r5.57.gff.gz' saved [377738814]
```

Subsequently unzipped the file and extracted all genes using the following command - extracting only the GFF records which have the word "gene" inbetween two spaces

```
In [46]: !gunzip dmel-all-r5.57.gff.gz
         !egrep "\tgene\t" dmel-all-r5.57.gff > dmel.57.gene.gff
```

To remove all miRNA and snoRNA records (assuming we are only interested in protein coding genes):

```
In [15]: !grep -v ' mir-'  dmel.57.gene.gff  | grep -v ' snoRNA'   ₩
             > dmel.57.gene.norg.gff
         !wc -l dmel.57.gene.norg.gff
```

```
16771 dmel.57.gene.norg.gff
```

Similarly - to extract all coding (parts of) exons:
Now loading these files into memory.

```
In [12]: #loading the gff with protein coding genes
         gff_cols = [' chr' , ' src' , ' type' , ' start' , ' stop' ,
                     ' score' , ' strand' , ' frame' , ' attr' ]

         gff_gene = pd.read_csv("dmel.57.gene.norg.gff", sep="₩t", names=gff_cols)
         #convert start & stop to integers (not certain if this is necessary)
         gff_gene[' start' ] = gff_gene[' start' ].map(lambda x: int(x))
         gff_gene[' stop' ] = gff_gene[' stop' ].map(lambda x: int(x))

         #extract the gene name from the attribute list
         gff_gene[' gene' ] = gff_gene[' attr' ].map(lambda x:x.split(' ;' )[1].split(' =' )[1])

         #how many genes do we have?
         print len(gff_gene)
```

```
16771
```

## 1.5 Map genes to MiMIC elements

A function (registerDist) is defined to assess and register for each gene which MiMIC element is nearby.

```
In [13]: def registerDist(distance,  gene, gchrom, gstart,
                                     gstop, column, touch_or_include="touch"):
             gstart, gstop = sorted([gstart, gstop])
             # ' touch'  means - 70k touches an element
             if touch_or_include == "touch":
                 # the element is on the same chromosome
                 # AND MIS + distance is bigger than gene/cds start
                 # AND MIS - distance is smaller than gene/cds stop
                 milist[ (mchrom == gchrom) &
                         (mpos >= gstart - distance) &
                         (mpos <= gstop + distance)
                       ][column].map(lambda  x:x.add(gene))
             else:
             # ' within'  means gene is fully within 70k
                 milist[ (mchrom == gchrom) &
                         (mpos >= gstop - distance) &
                         (mpos <= gstart + distance)
                       ][column].map(lambda  x:x.add(gene))
```

```
In [14]: # Map genes to the MiMic elements with differing window sizes
         for distance in [0, 10, 20, 30, 35, 40, 50, 60, 70, 80, 90, 100]:
             # create two emtpy fields to contain the genes that either
             # touch the ' distance'  window, and are completely within.
             milist[' gene_touch_%dk'  % distance] = ₩
                 [ set() for i in range(len(milist))]
             milist[' gene_within_%dk'  % distance] = ₩
```

```
            [set() for i in range(len(milist))]

        # Map gene/cds to mitos elements within/touch
        gff_gene.apply(lambda  x: registerDist(
                            distance * 1000,
                            x.name, x['chr'],
                            x['start'], x['stop'],
                            'gene_touch_%dk' % distance,
                            "touch"),
                    axis=1)
        gff_gene.apply(lambda  x: registerDist(
                            distance * 1000,
                            x.name, x['chr'],
                            x['start'], x['stop'],
                            'gene_within_%dk' % distance,
                            "within"),
                    axis=1)
```

```
In [16]: # Count how many genes are in the neighbourhood of each
         # MiMIC element
         for x in [0, 10, 20, 30, 35, 40, 50, 60, 70, 80, 90, 100]:
             milist['gene_count_within_%dk' % x] = ₩
                 milist['gene_within_%dk' % x].map(lambda x: len(x))
             milist['gene_count_touch_%dk' % x] = ₩
                 milist['gene_touch_%dk' % x].map(lambda x: len(x))
```

### 1.5.1 Export to disk

```
In [17]: milist.to_csv('MiMIC_to_gene.tsv', sep="₩t", index=False)
```

### 1.5.2 Summarize results

```
In [18]: results = pd.DataFrame(
             index=[0, 10, 20, 30, 35, 40, 50, 60, 70, 80, 90, 100])
         results['gene_within'] = [0] * 12
         results['gene_touch'] = [0] * 12
```

```
In [19]: #calculate numbers of unique genes per category
         def get_unique_sum(cat):
             allgenes = set()
             milist[cat].map(lambda x: allgenes.update(x))
             return len(allgenes)

         for x in [0, 10, 20, 30, 35, 40, 50, 60, 70, 80, 90, 100]:
             for c2 in ['within', 'touch']:
                 results['gene_%s' % (c2)][x] = get_unique_sum('gene_%s_%dk' % (c2, x))
                 results['gene_%s_frac' % (c2)] = results['gene_%s' % c2] / float(len(gff_gene))

         print results
```

| | gene_within | gene_touch | gene_within_frac | gene_touch_frac |
|---|---|---|---|---|
| 0 | 0 | 3316 | 0.000000 | 0.197722 |
| 10 | 9784 | 12270 | 0.583388 | 0.731620 |
| 20 | 13618 | 14824 | 0.811997 | 0.883907 |
| 30 | 15088 | 15686 | 0.899648 | 0.935305 |

| | | | | |
|---|---|---|---|---|
| 35 | 15471 | 15908 | 0.922485 | 0.948542 |
| 40 | 15710 | 16051 | 0.936736 | 0.957069 |
| 50 | 16033 | 16236 | 0.955995 | 0.968100 |
| 60 | 16220 | 16357 | 0.967146 | 0.975315 |
| 70 | 16333 | 16417 | 0.973883 | 0.978892 |
| 80 | 16391 | 16453 | 0.977342 | 0.981039 |
| 90 | 16437 | 16482 | 0.980085 | 0.982768 |
| 100 | 16470 | 16500 | 0.982052 | 0.983841 |

In [31]:
```
%matplotlib inline
plt.figure(figsize=(10,6))
plt.plot(results.index, results['gene_touch_frac'], label="gene touch")
plt.plot(results.index, results['gene_within_frac'], label="gene within")
plt.xlabel("Distance to the MiMic element (kb)")
plt.ylabel("Fraction of genes")
plt.vlines(70, 0, 1, label="70k")
plt.vlines(35, 0, 1, "green", label="35k")

plt.legend(loc=4)
plt.savefig("MiMic.take2.genes.vs.distance.png", dpi=300)
```

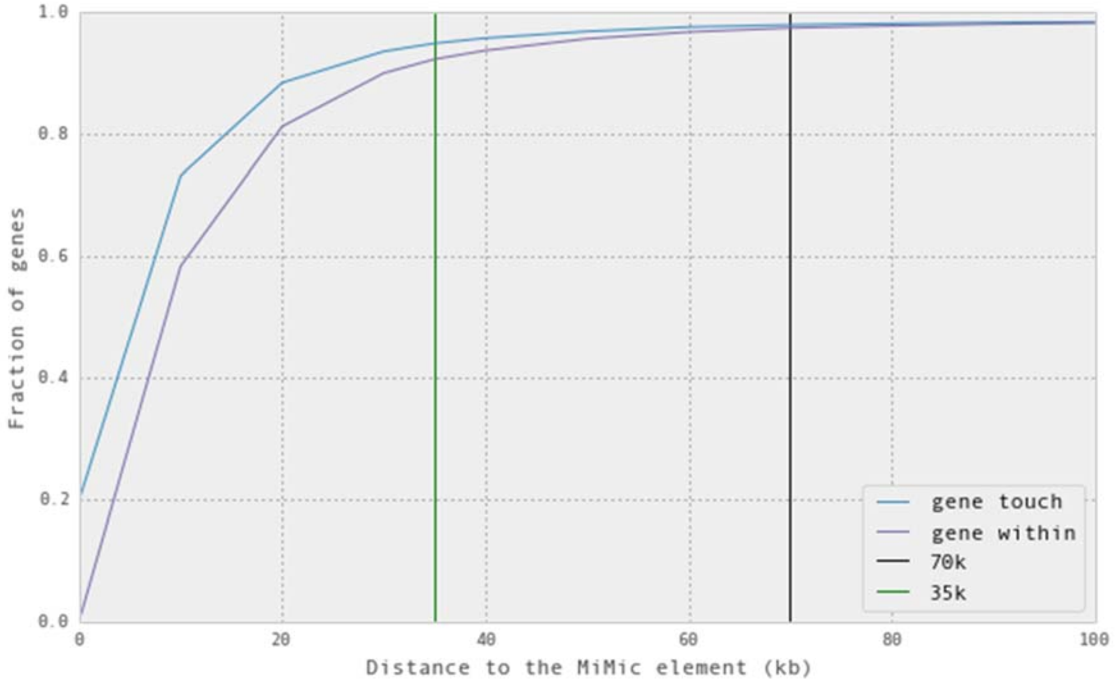


In [29]:
```
milist['genecount_within_70k'] = milist['gene_within_70k'].map(len)
x = milist['genecount_within_70k'].hist(bins=range(0,100,5))
x.set_xlabel("No of genes within 70k of a MiMic Element")
x.set_ylabel("No of MiMic elements")
x.set_xlim(0, 100)
plt.savefig("MiMICDistributionGene.70k.png", dpi=300)
```

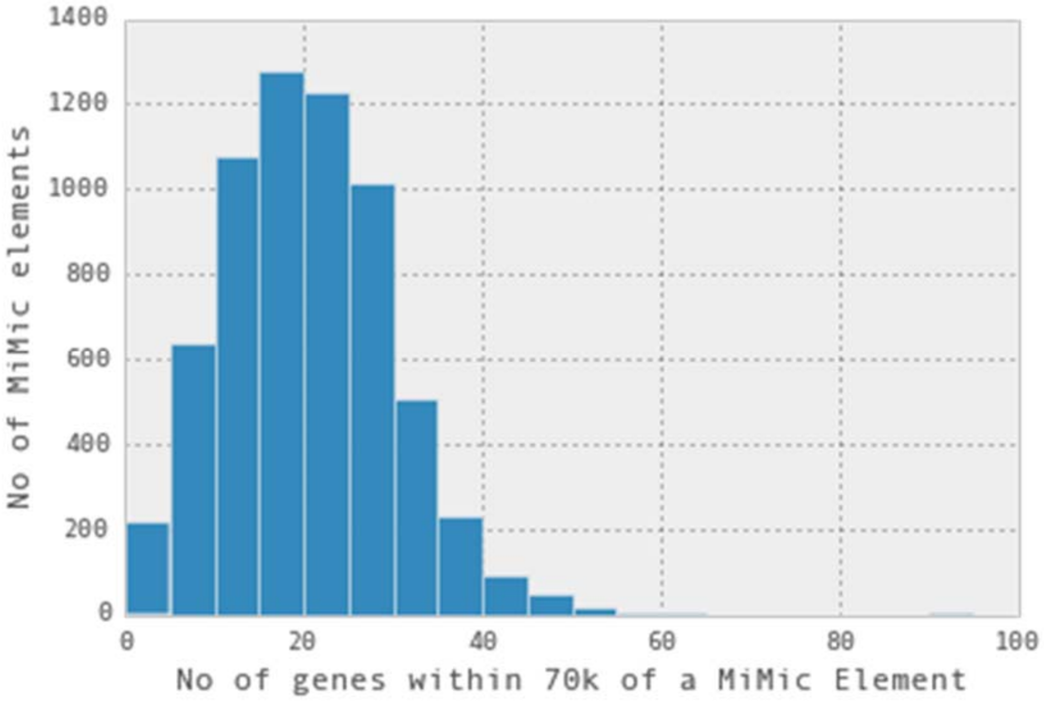


```
In [30]: milist['genecount_within_35k'] = milist['gene_within_70k'].map(len)
         x = milist['genecount_within_35k'].hist(bins=range(0,100,5))
         x.set_xlabel("No  of genes within 35k of a MiMic Element")
         x.set_ylabel("No  of MiMic elements")
         x.set_xlim(0,  100)
         plt.savefig("MiMicDistributionGene.35k.png", dpi=300)
```

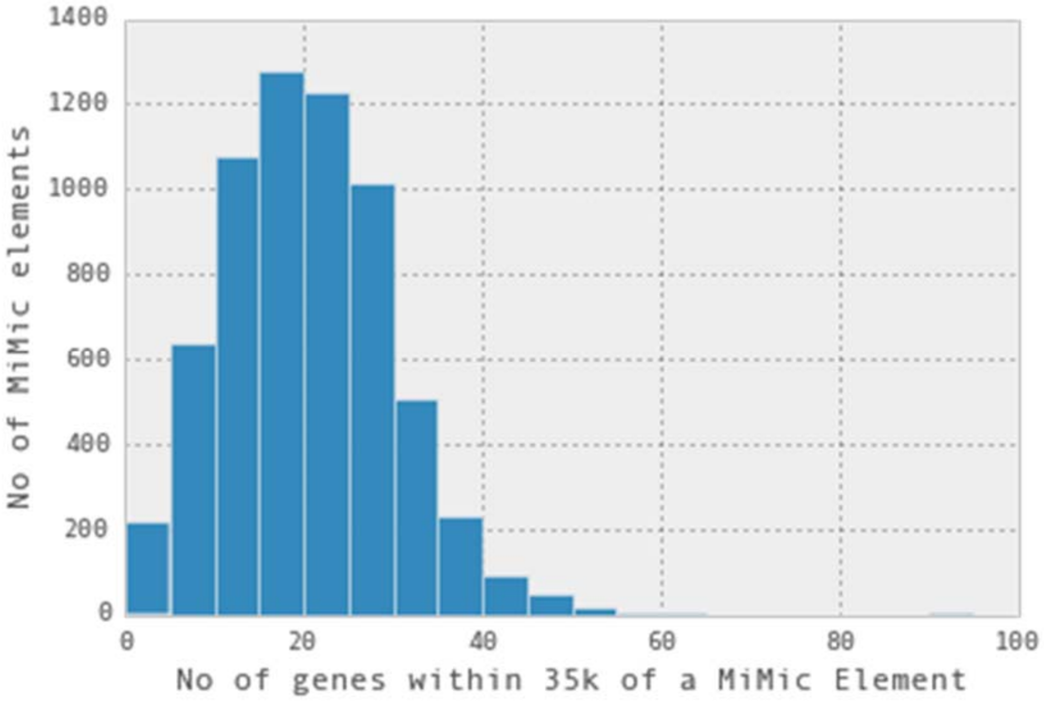

No of MiMic elements
1400
1200
1000
800
600
400
200
0
0
20
40
60
80
100
No of genes within 35k of a MiMic Element
