# Supplementary material for: Fast and Efficient Drosophila melanogaster Gene Knock-Ins Using MiMIC Transposons
Source: G3 (Bethesda). 2014 Oct 8;4(12):2381–7. doi: 10.1534/g3.114.014803 (PMC4267933; doi:10.1534/g3.114.014803)
Supplement: Supporting Information [file supp_g3.114.014803_FigureS1.pdf]

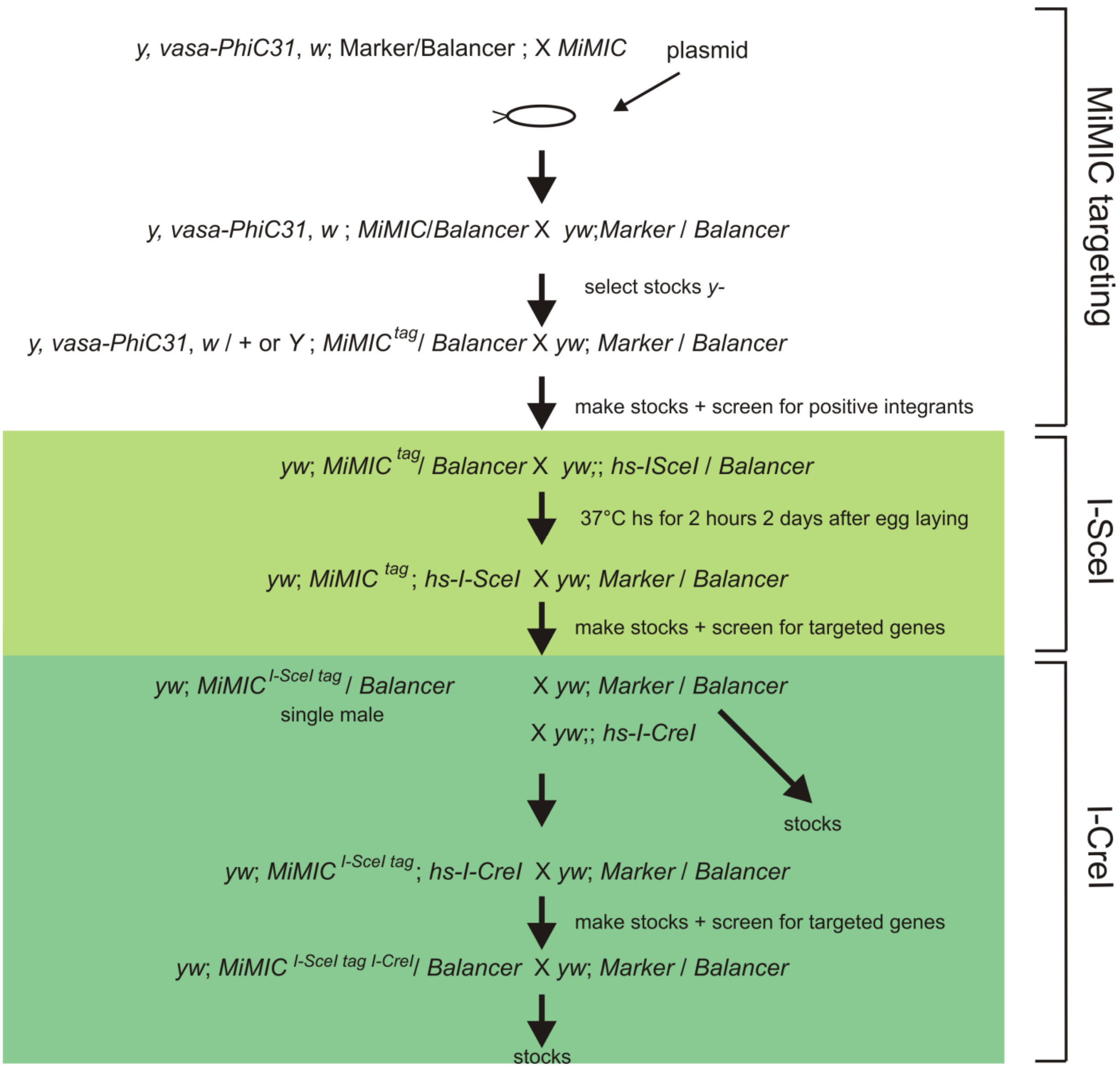

Figure S1 Overview of the crossing scheme used to create double strand breaks using restriction endonucleases I-SceI and I-CreI. During the MiMIC targeting step, the targeting plasmid is injected in embryos from flies bearing the MiMIC of interest (here for example on the 2nd chromosome) and the PhiC31 integrase. After crossing out these mosaic flies, screening for loss of yellow indicates the integration of the targeting construct in the MiMIC site. Next a stock is established and screening by PCR for integrants in the correct orientation is done. Upon I-SceI expression (light green) by crossing the *yw; MiMIC^{tag}/Balancer* stock to flies expression I-SceI under a heat shock promotor, a DSB will be generated after given a heat shock, followed by repair. After crossing out these mosaic flies the next generation will yield males that are non mosaic, since I-SceI is very efficient these males can be either used to set up stocks first or crossed simultaneous with flies that express I-CreI under heat shock promotor and flies that allow us to set up stocks. Upon I-CreI expression a DSB will be generated after a heat shock treatment, followed by repair. After crossing out these mosaic flies stocks can be set and screened for targeted genes by PCR. (dark green) (every arrow is one generation)
