# Supplementary material for: Fast and Efficient Drosophila melanogaster Gene Knock-Ins Using MiMIC Transposons
Source: G3 (Bethesda). 2014 Oct 8;4(12):2381–7. doi: 10.1534/g3.114.014803 (PMC4267933; doi:10.1534/g3.114.014803)
Supplement: Supporting Information [file supp_g3.114.014803_FigureS2.pdf]

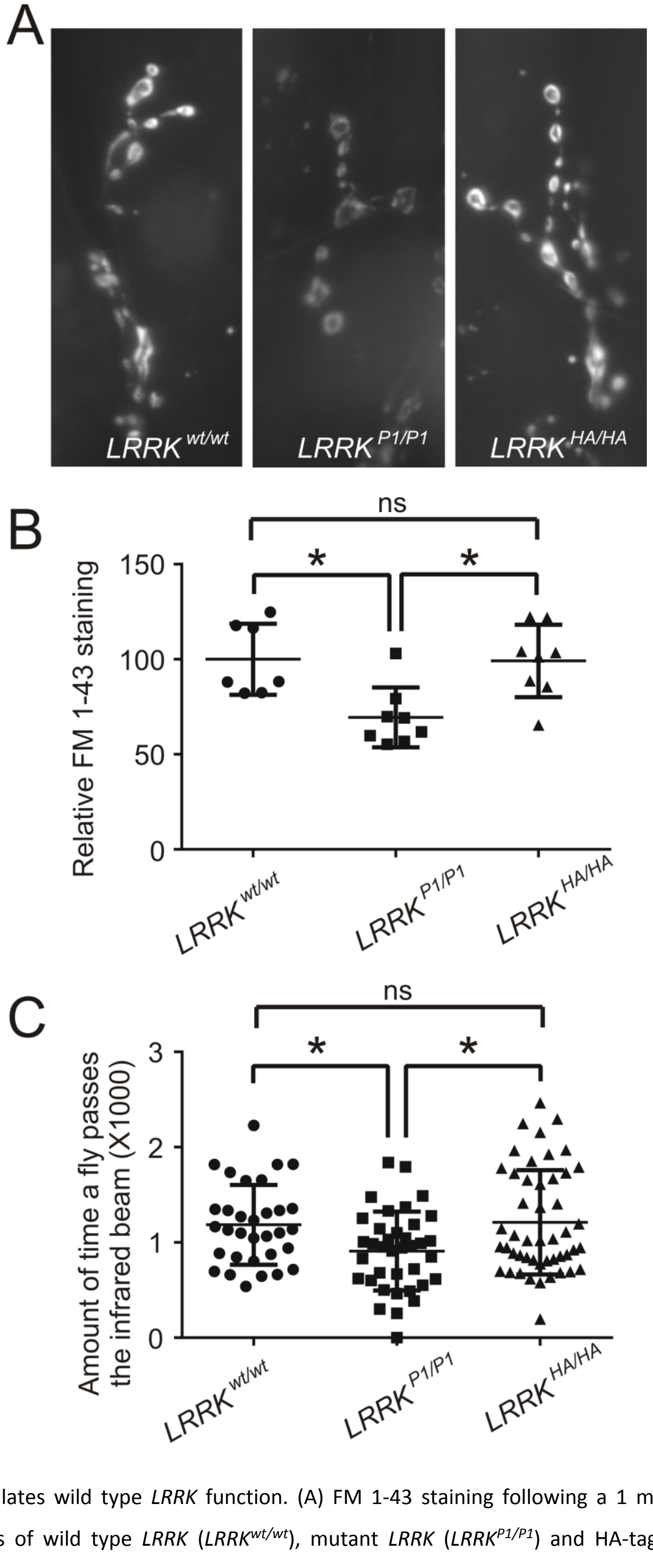

Figure S2 *LRRK*^{HA} recapitulates wild type *LRRK* function. (A) FM 1-43 staining following a 1 min 90 mM KCl stimulation protocol in larval boutons of wild type *LRRK* (*LRRK*$^{wt/wt}$), mutant *LRRK* (*LRRK*$^{P1/P1}$) and HA-tagged *LRRK* (*LRRK*$^{HA/HA}$), (B) indicating a defect in FM 1-43 dye uptake in mutant *LRRK* ^{P1} but not in HA-tagged *LRRK*^{HA} compared to wild type. Thereby showing reduced synaptic vesicle endocytosis in *LRRK* ^{P1}, not present in HA-tagged *LRRK*^{HA}. (C) Trikinetics activity assay indicating total activity counts over a 24 h period of the same genotypes as in (A) en (B). A defect in total activity in mutant *LRRK* ^{P1} but not in HA-tagged *LRRK*^{HA} compared to wild type *LRRK* is evident.
