# Supplementary material for: Fast and Efficient Drosophila melanogaster Gene Knock-Ins Using MiMIC Transposons
Source: G3 (Bethesda). 2014 Oct 8;4(12):2381–7. doi: 10.1534/g3.114.014803 (PMC4267933; doi:10.1534/g3.114.014803)
Supplement: Supporting Information [file supp_g3.114.014803_FigureS3.pdf]

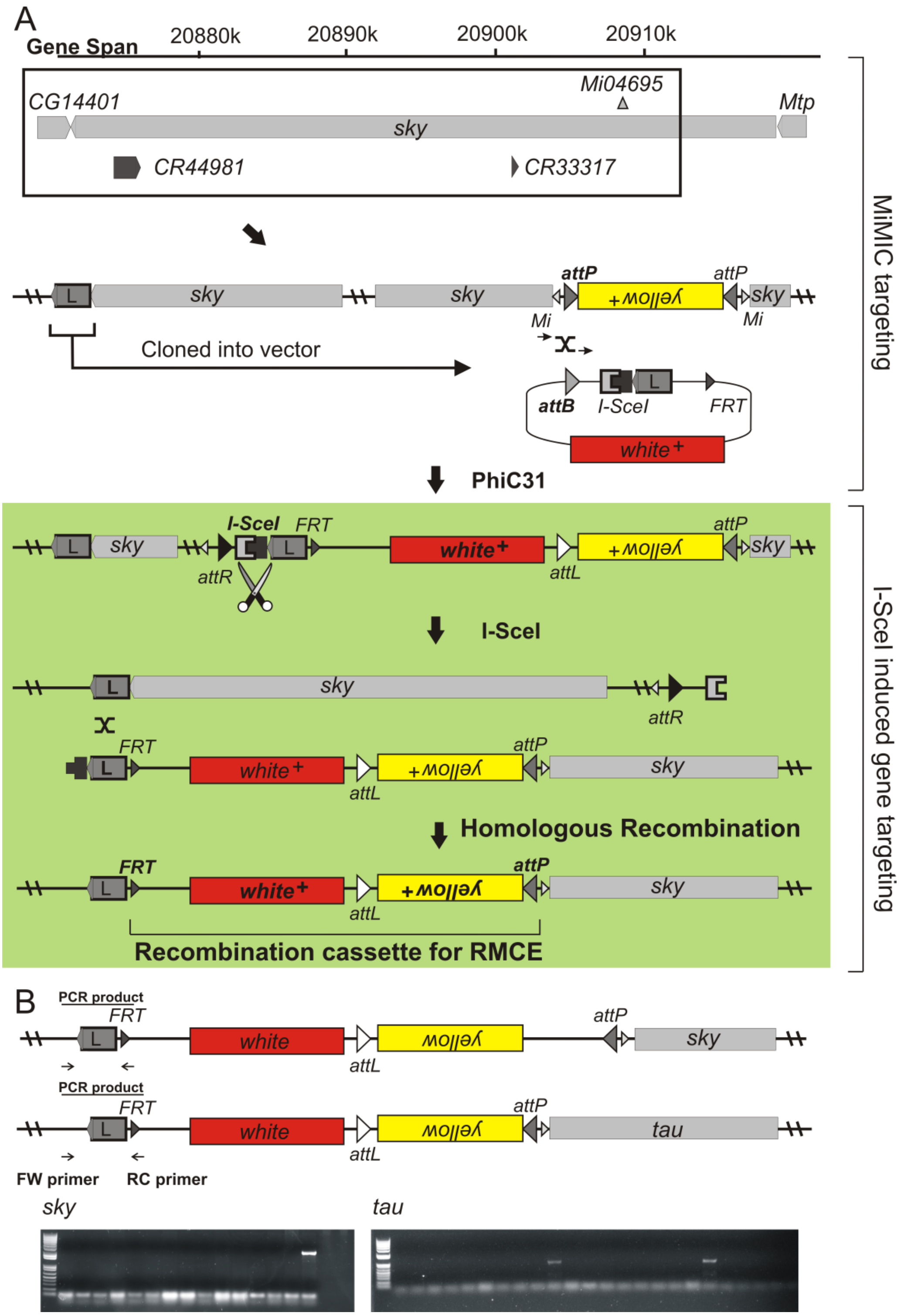

Figure S3 Strategy for *sky* targeting and molecular confirmation of targeted *sky* and *tau* loci. (A) Targeting strategy *sky* with an RMCE cassette using a MiMIC in *sky*. *I-SceI* expression followed by ssa results in a partial replacement of *sky* with an RMCE thereby deleting the majority of the sky coding sequences (green). (B) Schematic representation showing where primers (arrows) anneal to generate PCR products, one primer anneals outside the homology arm, the other anneals in the RMCE cassette. Representative agarose gels are shown, a PCR band indicates a successful targeting. Bold text marks which sites/enzymes/mechanisms are being used. Green color indicating I-SceI induced gene targeting match in crossing scheme (Figure S4).
