# Supplementary material for: Fast and Efficient Drosophila melanogaster Gene Knock-Ins Using MiMIC Transposons
Source: G3 (Bethesda). 2014 Oct 8;4(12):2381–7. doi: 10.1534/g3.114.014803 (PMC4267933; doi:10.1534/g3.114.014803)
Supplement: Supporting Information [file supp_g3.114.014803_FigureS4.pdf]

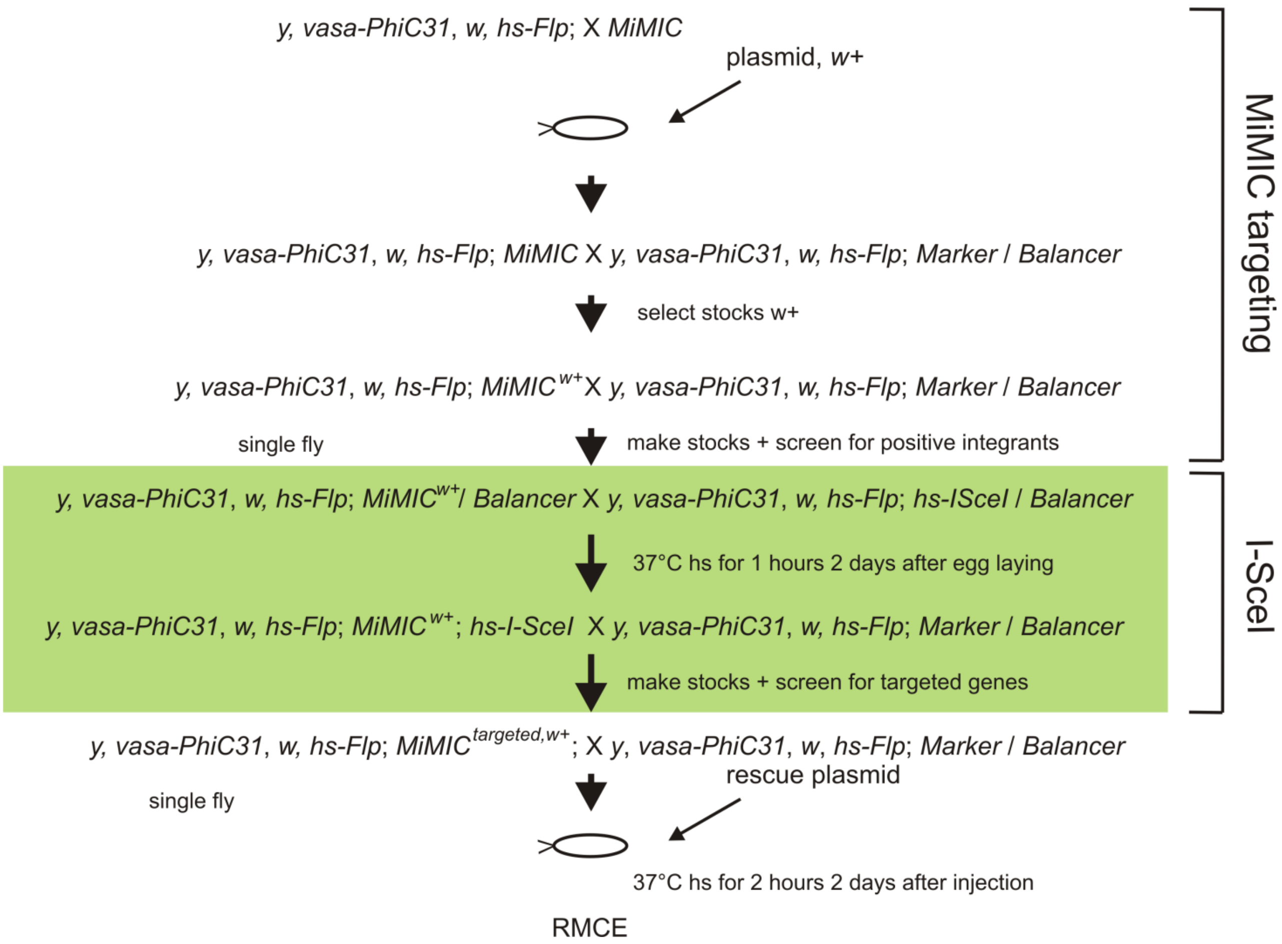

Figure S4 Crossing scheme for direct targeting of a gene of interest (here on the 2nd chromosome) using a MiMIC transposon. During the MiMIC targeting step, the targeting plasmid is injected in embryos from flies bearing the MiMIC of interest and the PhiC31 integrase. After crossing out these mosaic flies, screening for w^+ indicates the integration of the targeting construct in the MiMIC site. Next a stock is established and screening by PCR for integrants in the correct orientation is done. Crossing the *yw; MiMIC^{w+}/Balancer* stock to flies expressing I-SceI under a heat inducible promotor allows us to generate a DSB after heat shock, followed by repair. After crossing out these mosaic flies a stock is made from the offspring and screened by PCR for targeted events (green). Positive stocks replaced the gene of interest by an RMCE cassette, which in a next step can be used to introduce any construct of choice including rescue constructs via PhiC31 integration and Flp. Arrows indicate one generation.
