# Supplementary material for: Fast and Efficient Drosophila melanogaster Gene Knock-Ins Using MiMIC Transposons
Source: G3 (Bethesda). 2014 Oct 8;4(12):2381–7. doi: 10.1534/g3.114.014803 (PMC4267933; doi:10.1534/g3.114.014803)
Supplement: Supporting Information [file supp_g3.114.014803_FigureS5.pdf]

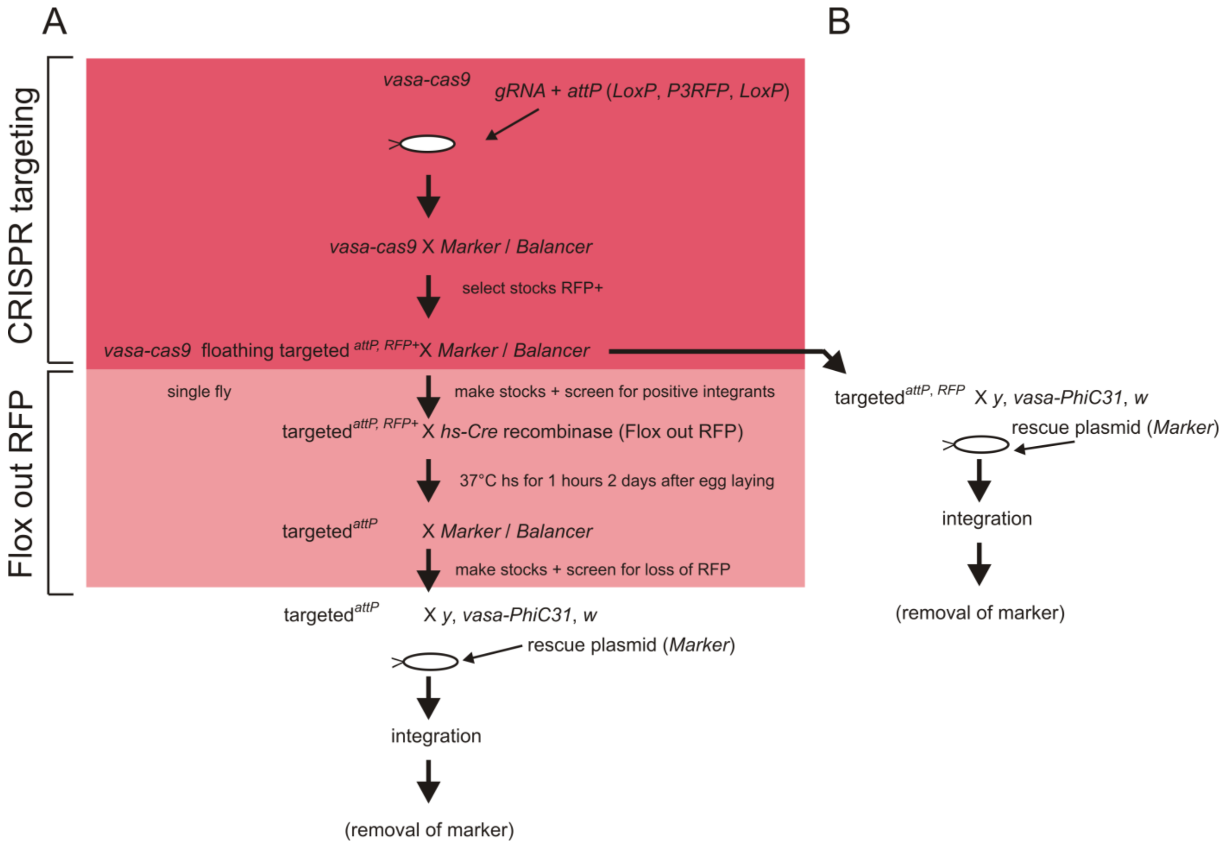

Figure S5 Crossing scheme for gene targeting using CRISPR/Cas9. (A) CRISPR targeting by injecting gRNA and the targeting construct bearing an attP site and P3 RFP marker flanked by LoxP sites, in vasa-Cas9 embryos, followed by crossing out mosaic flies and selection for RFP (dark pink). Next the RFP marker can be floxed out by crossing the targeted flies to flies expressing the Cre recombinase under a heat shock promotor, followed by crossing out mosaic flies and screening for the loss of RFP (light pink). Stocks that lost the RFP marker, bearing the attP site can in a next step be targeted with a rescue plasmid (preferentially with a marker) via PhiC31 integration. (B) Or after CRISPR targeting, single flies can be targeted with a rescue plasmid (preferentially with a marker) without first removal of the RFP marker, however these markers need to be removed afterwards. Arrows indicate one generation.
